# Locomotion Induced by Spatial Restriction in Adult *Drosophila*

**DOI:** 10.1371/journal.pone.0135825

**Published:** 2015-09-09

**Authors:** Chengfeng Xiao, R. Meldrum Robertson

**Affiliations:** Department of Biology, Queen’s University, Kingston, ON, K7L 3N6, Canada; Alexander Fleming Biomedical Sciences Research Center, GREECE

## Abstract

*Drosophila* adults display an unwillingness to enter confined spaces but the behaviors induced by spatial restriction in *Drosophila* are largely unknown. We developed a protocol for high-throughput analysis of locomotion and characterized features of locomotion in a restricted space. We observed intense and persistent locomotion of flies in small circular arenas (diameter 1.27 cm), whereas locomotion was greatly reduced in large circular arenas (diameter 3.81 cm). The increased locomotion induced by spatial restriction was seen in male flies but not female flies, indicating sexual dimorphism of the response to spatial restriction. In large arenas, male flies increased locomotion in arenas previously occupied by male but not female individuals. In small arenas, such pre-conditioning had no effect on male flies, which showed intense and persistent locomotion similar to that seen in fresh arenas. During locomotion with spatial restriction, wildtype Canton-S males traveled slower and with less variation in speed than the mutant w1118 carrying a null allele of *white* gene. In addition, wildtype flies showed a stronger preference for the boundary than the mutant in small arenas. Genetic analysis with a series of crosses revealed that the *white* gene was not associated with the phenotype of boundary preference in wildtype flies.

## Introduction

*Drosophila* adults display an unwillingness to enter confined spaces, which has been described as a claustrophobic effect [1]. When individual flies are restrained in a small rectangular arena (1.0 cm × 0.6 cm), locomotion is characterized by relentless or unabated activity for several hours, interspersed with a few short episodes of inactivity [2]. In a larger, circular arena (9.1 cm diameter), however, locomotor activity of individual flies can be distinguished as being reactive or as having a period of initial elevated activity in response to novel experimental situations followed by spontaneous activity at a steady rate [3–5]. The change from reactivity to spontaneous activity can be observed in relatively large but not small arenas, indicating that flies respond to arena size with different styles of locomotion.

Spatial restriction has diverse behavioral consequences in other animals. Confinement imposes movement limitations in egg-laying hens [6–8], and induces behavioral and psychological disorders in captive animals [9–12]. A common abnormality associated with spatial restriction is the occurrence of stereotypic behaviors–a group of repetitive and non-varying behavioral patterns [9], for instance, compulsive pacing in cats and bar gnawing in laboratory-caged mice [13, 14]. Such stereotypic behavior is an indication of suffering from the restricted environment [15]. Spatial restriction also increases timidity [16], aggression [17], hyperphagia and polydipsia [18] and extreme restraint induces gastric ulceration [19] and increases heart rates [20]. In *Drosophila*, flies with high reactivity show circling patterns of locomotion along the edge of a circular chamber with 2.54cm diameter, and keep their distance from each other [1]. In the open field, however, individual flies locomote with two main characteristics: directional persistence and wall attraction [21]. In circular arenas of 8.4, 9.1, 11.7 or 15.0 cm diameter, or in a square of 4.0 × 4.0 cm, freely-moving flies display wall or peripheral preference during locomotion [4, 22–25]. However, in a relatively large arena of 24.5 cm diameter, flies display a number of behavioral patterns including walking, stopping, sharp turns, crab-walking, backing up and jumping [26]. The behavioral performance of flies rigidly tethered with a metal wire [27–29] represents activity under extreme confinement and thus natural locomotor activities are limited. Although it has been implied that locomotion is different in specific spatial environments, not much is known about the effect of spatial restriction on the locomotion of *Drosophila* adults.

The wildtype strain Canton-S (CS) and the mutant w1118 that carries isogenic X, second and third chromosomes and a null allele of *white* (*w*) gene on the X chromosome [30–33] are widely used as controls in the *Drosophila* community. In addition, the majority of transgenic fly lines are generated using w1118 as genetic background. As a starting point to improve our understanding of fly behavior in confinement we explored the characteristics of locomotion with spatial restriction in CS and w1118 flies. We hypothesized that spatial restriction motivates persistent exploration to search for an escape. To characterize locomotion, we developed a protocol for large-scale analysis of locomotor activity in arenas with different sizes. Using this protocol, we identified several locomotor features associated with spatial restriction. Wildtype and mutant flies showed high sensitivity to spatial restriction, and strain-specific differences in step size and boundary preference with spatial restriction. We further demonstrated that the *w* gene was not associated with the high degree of boundary preference in wildtype flies.

## Materials and Methods

### Fly strains

Wildtype Canton-S and mutant w1118 flies were maintained on standard medium (supplied by L. Seroude laboratory) at room temperature (21–23°C) with 60–70% humidity. 12 h/12 h light/dark illumination was provided by three light bulbs (Philips 13 W compact fluorescent energy saver) with lights on and off at 7 am and 7 pm. All experiments were conducted in the daytime between 10 am and 4 pm. Adult flies were collected through nitrogen anesthesia within 2 days after eclosion and raised in fresh food vials for at least 3 days before locomotor assays. A minimal period of 3 days without anesthesia was guaranteed before experiments which used flies no more than 9 days old.

### Fly crosses

Three different crosses were performed between CS and w1118 flies. Simple crosses were between male w1118 and female CS, and reciprocally, between male CS and female w1118. These crosses generated F1 male flies with the same genetic content on chromosome II and III but different chromosome X and cytoplasmic background. Introgression [34] was performed by initially crossing female w1118 with CS male flies. The F1 female flies were backcrossed with CS males again. Backcrossing between female progeny and CS males was carried out three times. F2 to F4 males were separated as *w* ^+^ and *w* ^1118^-carrying groups and tested. The introgression was intended to generate fly lines carrying gradually increased chromosomal content of CS in w1118 cytoplasmic background. The tested fly groups (*w* ^+^ and *w* ^1118^-carrying males in the same generation) were from the same mothers and thus had synchronized genetic and cytoplasmic content excluding *w* alleles. Serial backcrossing was performed by initially crossing male CS or w1118 ancestor into female w1118 or CS to produce *w* ^+^/ *w* ^1118^ heterozygous female progeny. These progeny were then backcrossed with w1118 or CS strain. Backcrosses between *w* ^+^-carrying flies and w1118 strain and between *w* ^1118^-carrying flies and CS strain were carried out for a total of nine generations. The resulting *w* ^+^- and *w* ^1118^-carrying flies (F10) had *w* alleles in different genetic and cytoplasmic backgrounds from the original.

### Locomotor assay

**Fig 1:**
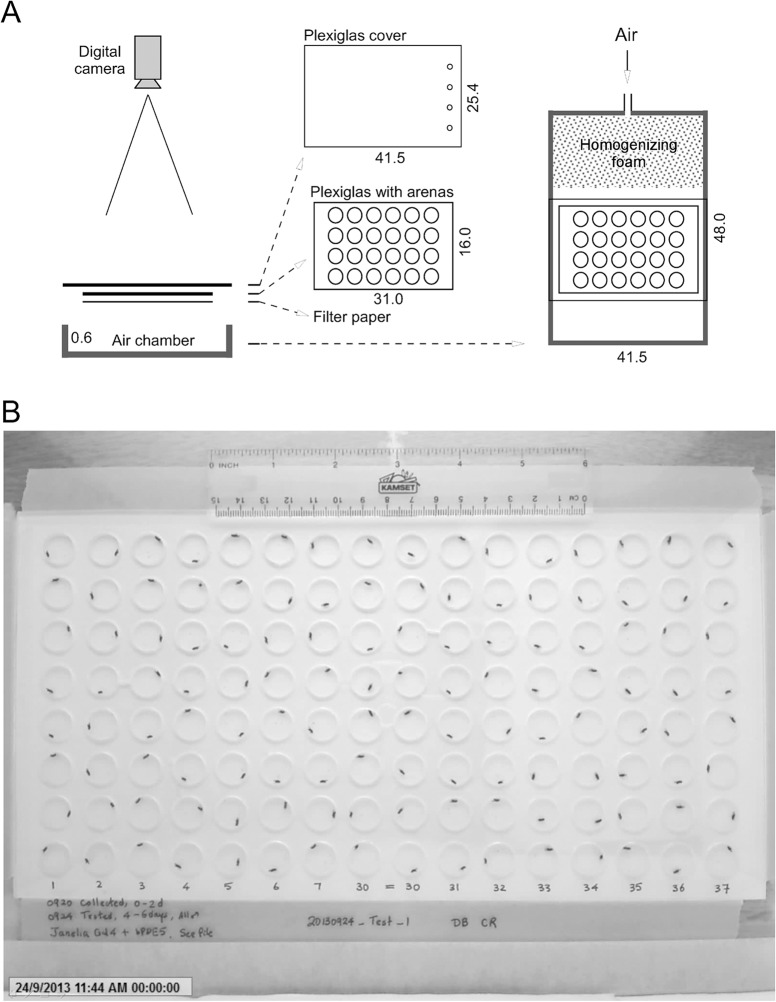
An apparatus for the locomotor assay. (A) A schematic drawing showing the experimental settings for the analysis of locomotor activities of *Drosophila* adults. A digital camera was linked to a computer and operated by associated software for video capture. A Plexiglas sheet with circular arenas was placed between a sliding Plexiglas cover and thick filter paper. Individual flies were loaded through holes (0.3cm diameter) on the side of plastic cover. Plexiglas sheets with different sized arenas were used for the analysis of locomotion with different spatial constraints. See supplementary information for more details. (B) The first video frame showing arenas (1.27cm diameter) loaded with flies and the experimental labels. Only one fly was loaded into each arena. The resolution of this image is 1600 × 1200 pixels. A total of 128 flies were loaded and analyzed in this video. A metric ruler with dimensions (cm) was placed along the sides for pixel-cm conversion.

An apparatus was constructed for high-throughput analysis of locomotion of multiple individual flies at the same time (Fig 1). An array of circular arenas (1.27 cm diameter) was machined in a 0.3 cm-thick Plexiglas sheet. The 0.3 cm thickness allowed flies to turn around and suppressed vertical movement. The bottom of the arenas was covered with chromatography paper (Cat# 05-714-4, Fisher Scientific) for air circulation. The top was covered with another slightly larger and sliding Plexiglas sheet with loading holes (0.3 cm diameter) close to one end. Flies were gently aspirated into arenas through the loading holes. Only one fly was loaded into each arena. Loading was performed column by column from left to right by sliding the cover. The assembled plate was then positioned in a larger air chamber. A slow air flow (2 L/min) was provided throughout the experiment to avoid the accumulation of aerobic or volatile metabolites such as CO_2_ (Fig 1A). The plate was illuminated with a white light box (Logan portaview slide/transparency viewer). Reflecting white cardboard sheets were used to improve lateral illumination, and for shielding the arenas from the experimenter and other activity in the room. Locomotor behavior of flies was video-captured with a digital camera (Logitech Webcam C905) and its associated software. The optical distortion measured from the center to the corner in an image was 0.7%. The distortion is negligible with respect to the image resolution of 1600×1200 pixels, and the fly size of 90–110 square pixels. The grey-scale videos (~71 min), formatted as Windows Media Video (.WMV) at a frame rate of 15 fps were taken and stored for later analysis on a frame-by-frame basis (Fig 1B). Experimental settings including illumination, light reflection, and camera configuration were maintained constant in different experiments.

### Arena pre-conditioning

Newly assembled arenas were loaded with individual male or female flies for 75–90 min at room temperature. The flies were then removed. These pre-conditioned arenas were re-loaded with other individual flies within 1h for locomotor analysis. Only virgin flies of the same strain with the same age as the tested flies were used for arena pre-conditioning.

### Fly tracking

A script was developed to calculate fly position (the center of mass) using free software: open computer vision 2.0 (OpenCV2.0) and Microsoft Visual C++ 2008 Express. The main procedures included, (i) learning a background from multiple frames of a video; (ii) comparing the difference between each frame and the background; (iii) computing the center of mass of each fly; and (iv) calculating path length over a period of time (e.g. 1 s). Fly positions were computed once every 0.2 s. A metric ruler was placed in the camera view alongside the arenas for pixel-mm conversion. The computed positional information was processed for parameter calculation in Excel. Further description of scripting is provided in the supporting information (S1 Text; S1 Appendix).

### Estimation of percentage of time on the perimeter

In the analysis of fly position either on or off the perimeter, the size of individual fly was evaluated by the software. The rationale was that, considering the substantial size difference between male and female, and probably between mutant and wildtype, a criterion using a uniform dimension may be unsuitable for determining whether a fly was on or off the perimeter. Therefore, the size specific to each fly was estimated. Once the fly size was obtained, a circular border surrounding the perimeter zone with the distance of a half fly size from the edge was set as a standard to differentiate the fly locations. If the center of a fly was on the standard border or within the area between edge and the standard, it was judged that the fly was on the perimeter. The percentage of time on the perimeter over a period of 60 s was evaluated as, % TOP = (numbers of position on the perimeter / total numbers of locations evaluated) × 100%. Positional information was evaluated once every 0.2 s. See supporting information (S1 Text; S1 Appendix) for more detail.

### Statistics

Sample sizes for each test were: n = 9 for large arenas, and n = 8 for small arenas, unless otherwise stated. A minimum of three replications for each experiment were conducted. Linear regression was performed to examine whether the velocity increased or decreased over 60 min. Specifically, linear regression was to determine whether the slopes of velocity over time in multiple flies were non-zero. A repeated measures ANOVA was used to compare the velocity during 60 min of locomotion between different flies or arenas. A histogram analysis (with bins of 0.02 cm/0.2s) was performed to examine the relative frequency (%) of path length per 0.2 second (the minimal interval for position computing) during 60 s of locomotion. Normality test was performed to examine whether the data follow Gaussian distribution. Nonparametric tests (Mann-Whitney test) were used to examine the difference of median path length per 0.2 s and variance during the 60 s locomotion between fly strains. Specific statistics were indicated in the text or figure legend. *P* < 0.05 was considered as significant difference between groups.

## Results

### Spatial restriction induced intense and persistent locomotion in male flies

**Fig 2:**
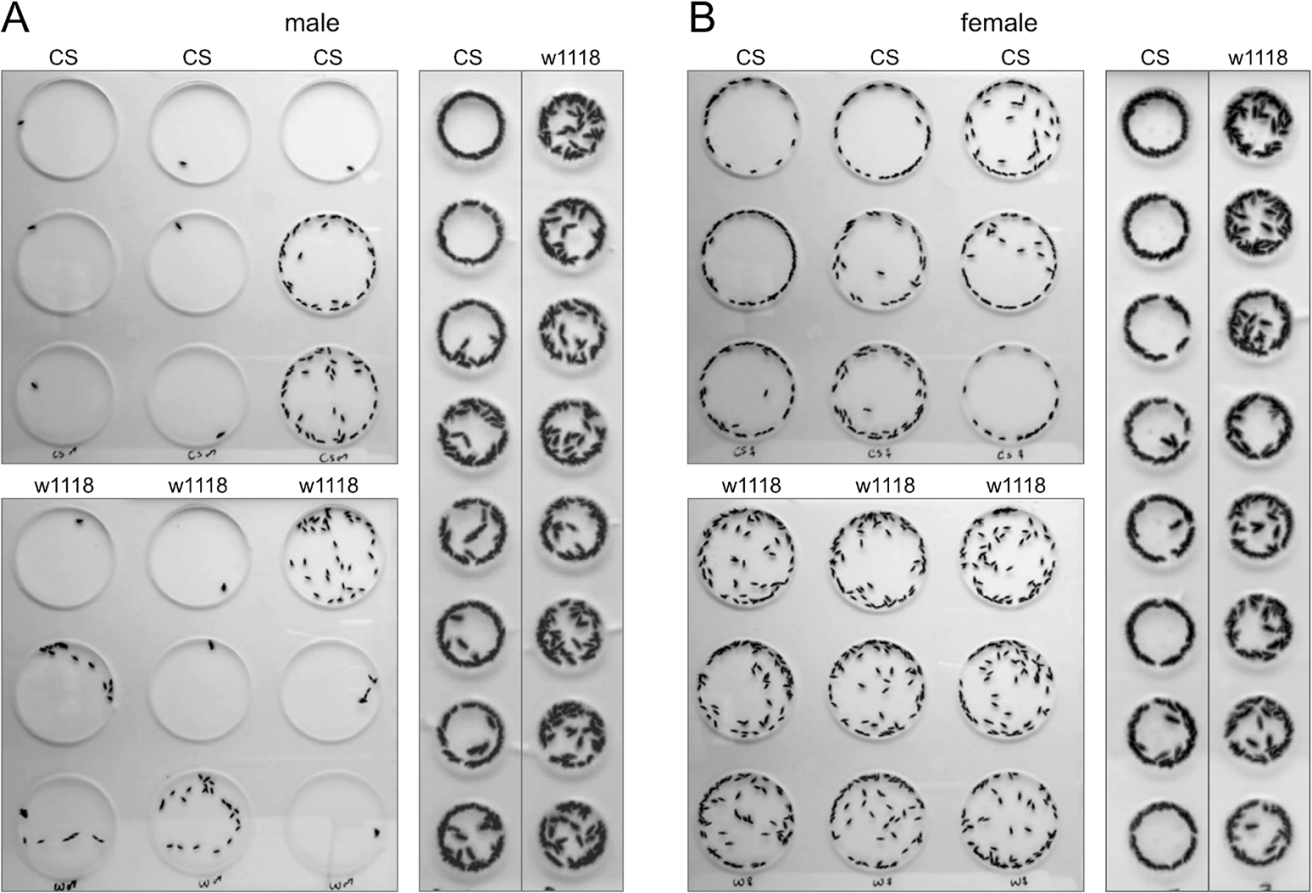
Locomotion in large and small arenas. Composite images of 60 overlapped frames of (A) male CS and w1118 flies, and (B) female CS and w1118 flies, extracted from 4th min of the videos. Each arena holds only a single fly so the different locations in the arenas indicate the activity of individuals during one minute.

Following a 5 min acclimation period, most CS male flies became inactive in the large circular arenas. During the 4th minute of recording, visually analyzed by overlapping 60 video frames, seven out of nine CS males did not substantially change their positions in the arenas, and only two flies continued to move (Fig 2A upper left). Similarly, four w1118 flies did not change their positions within a minute and two others showed minimal movement (Fig 2A lower left). In small circular arenas, however, all the CS or w1118 males showed continuous movement with frequent changes in position (Fig 2A right panel).

CS female flies changed locations continuously in large arenas after 5 min of acclimation. All the flies were active in locomotion and no flies remained motionless for a minute (Fig 2B upper left). Similarly, w1118 females walked continuously in large arenas. No individual was motionless during the 1 min of video recording (Fig 2B lower left). In small arenas, CS and w1118 female flies changed location continuously and remained active in locomotion similar to male flies in small arena (Fig 2B right).

**Fig 3:**
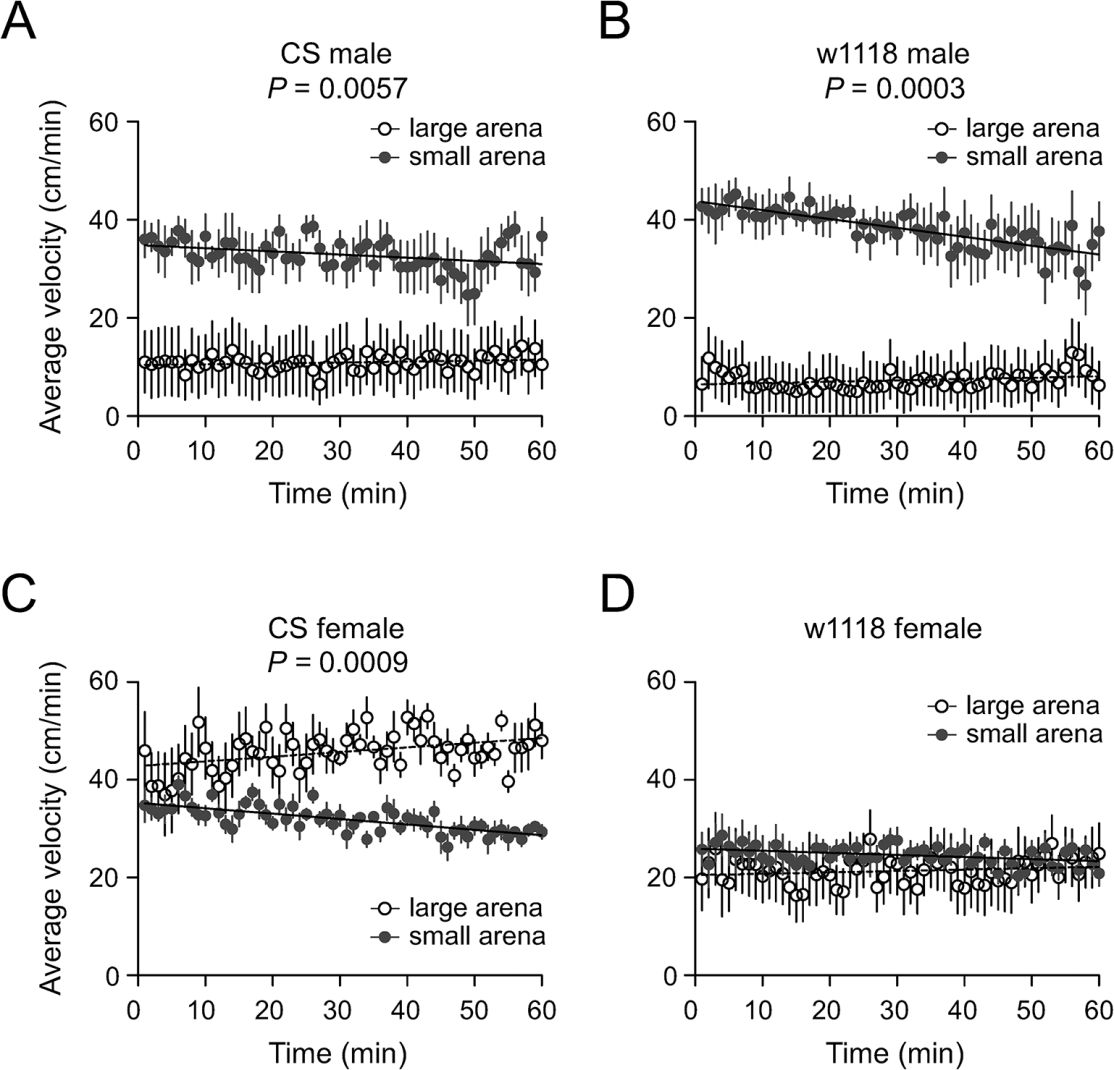
Analysis of locomotor activity. Plots of velocity (cm/min) versus time (min) for 60 min activities in large (open circles) and small arenas (solid circles) for: (A) CS male; (B) w1118 male; (C) CS female; and (D) w1118 female. Symbols and lines indicate means ± SE (n = 9 for large arenas; n = 8 for small arenas). Linear regression lines have been superimposed on the data to show the trend of locomotor velocity over time. *P* values indicate statistical comparison between large and small arenas. Only *P* values <0.05 are shown.

In large arenas CS males (n = 9) traveled initially with an average velocity of 11.0 cm/min, maintained for 1h without apparent change, whereas in small arenas CS males (n = 8) moved with a velocity >30 cm/min initially and maintained similarly high velocity for the duration of the experiment, though there was a small but significant decline during the hour (*P* < 0.05, linear regression). The velocities during 60 min locomotion in small arenas were greatly increased compared with those in large arenas (*P* = 0.0057, repeated measures ANOVA) (Fig 3A). In large arenas w1118 male flies (n = 9) traveled with an average velocity of 6.5 cm/min at the beginning and the flies maintained similar velocity for 1 h with small increase (*P* < 0.05, linear regression), whereas in small arenas w1118 males (n = 8) traveled at >40 cm/min initially and maintained a high velocity for 1 h with a slight and significant decline over time (*P* <0.05, linear regression). The velocities of w1118 during 60 min activity in small arenas were higher than those in large arenas (*P* = 0.0003, repeated measures ANOVA) (Fig 3B). Thus the reduction of arena size induced intense and persistent locomotion of both CS and w1118 male flies.

### Sexual dimorphism of locomotion with spatial restriction

Sexually dimorphic behaviors in mating, aggression and locomotion are common in flies [35–37]. In large arenas, CS females (n = 9) traveled initially at an average velocity around 45.9 cm/min and continued locomotion for 1 h with a small increase over time (*P* <0.05, linear regression). Compared with mostly inactive CS males in large arenas, female flies were markedly active. In small arenas, CS females moved at around 35 cm/min and maintained locomotion for 1 h with a gradual and significant decline (*P* <0.05, linear regression). The velocities in small arenas was less than that in large arenas (*P* = 0.0009, repeated measures ANOVA) (Fig 3C). Thus, CS males and females displayed different levels of locomotion in large arenas and opposite changes of locomotion as arena sizes reduced.

In large arenas w1118 females traveled at around 20 cm/min and maintained similar velocity for 1 h with no change. Compared with the inactive state of males in large arenas, females were highly active. In small arenas w1118 females moved initially at 25.8 cm/min and maintained locomotion with a slow decrease in velocity over time (*P* <0.05, linear regression). There was no difference of median velocity of w1118 females in large and small arenas (Fig 3D). Therefore, w1118 female flies displayed different levels of locomotion from males in large arenas. Specifically, w1118 female flies maintained the same level of locomotion, whereas w1118 male flies greatly increased locomotion as arena size reduced.

Taken together, in response to spatial restriction CS and w1118 male flies greatly increased locomotion, whereas CS female flies reduced and w1118 females maintained the same level of locomotion. These data indicate the sexually dimorphic nature of locomotion in CS and w1118 flies with the reduction of arena size.

### High sensitivity to spatial restriction in male flies

In a chamber of similar size to the small arenas used in our study, and in the presence of one or two virgin females, a male fly performs active courtship behavior to female fly [38, 39] rather than persistent locomotion. This suggests that locomotion with spatial restriction could be modified if the arenas are pre-conditioned by factors such as the presence of females or pre-occupancy by other individuals. We pre-conditioned the arenas and examined the sensitivity of male flies to spatial restriction.

**Fig 4:**
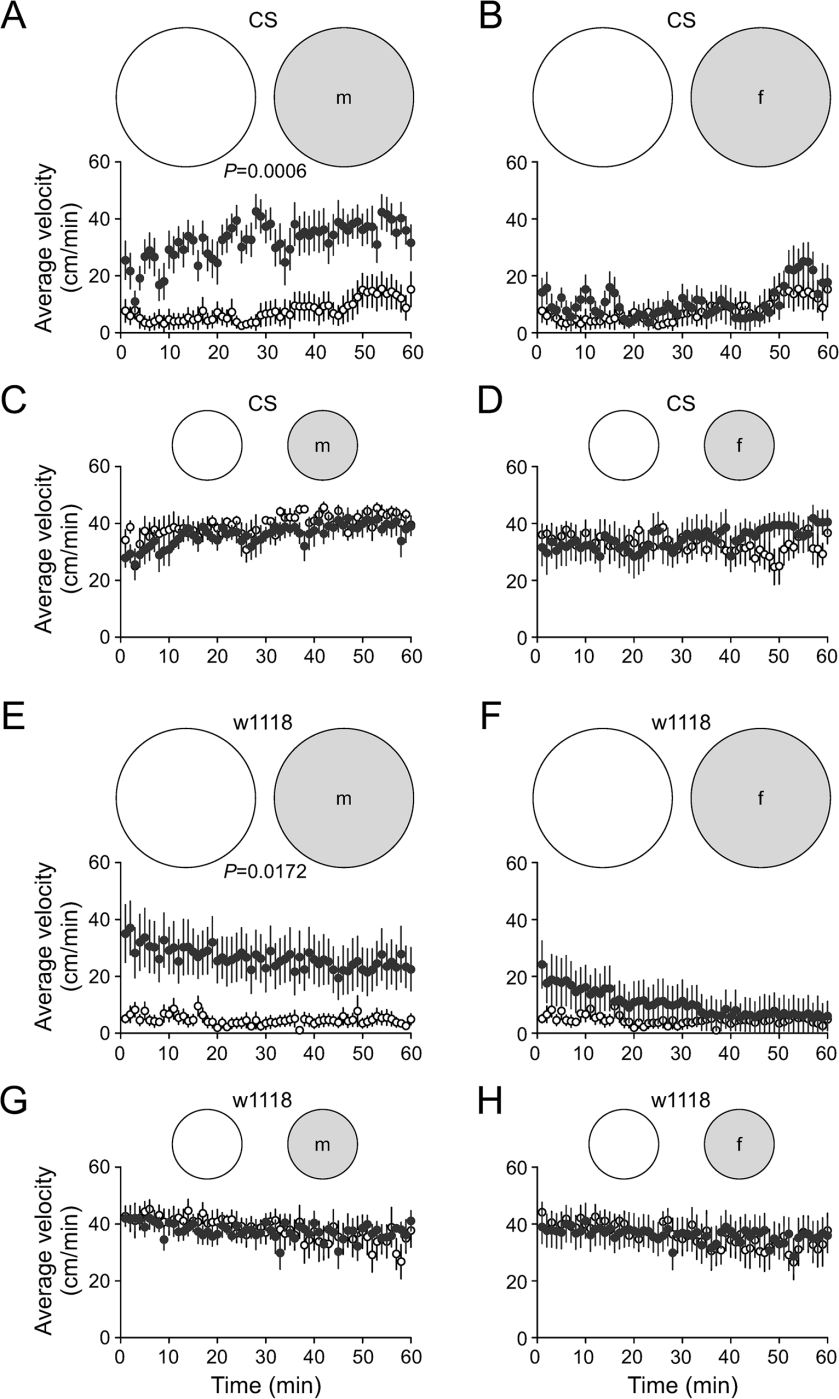
Locomotor activity in fresh and pre-conditioned arenas. Plots show the velocity (cm/min, means ± SE) of flies during 60 min activity. (A, B) Locomotion of male CS flies (n = 9) in large and fresh (blank circles) and pre-conditioned arenas (filled circles) which had been previously occupied by individual CS male (m) or female (f) fly. (C, D) Locomotion of male CS flies (n = 8) in the same conditions as A, B but in small arenas. (E, F) Locomotion of male w1118 flies (n = 9) in large and fresh and pre-conditioned arenas by w1118 male (m) or female (f). (G, H) Locomotion of male w1118 flies (n = 8) in the same conditions as E, F but in small arenas. Note: Pre-conditioning duration was 75–90 min by virgin flies of the same strain.

The pre-conditioning was performed by loading the arenas with individual virgin male or virgin female flies for a period of 75–90 min. Pre-conditioned arenas were emptied and reloaded with male flies of same strain within 1 h for locomotor assays. In large arenas, CS males traveled at a velocity of 7.7 cm/min and maintained a relatively steady locomotion for 60 min in fresh arenas. However, flies traveled at a much higher velocity of 20–40cm/min for the duration of the assay in male pre-conditioned arenas. The velocities of CS males during 60 min locomotion in male pre-conditioned arenas were markedly higher than those in fresh arenas (*P* = 0.0006, repeated measures ANOVA) (Fig 4A). In female pre-conditioned arenas, CS male flies traveled at velocities similar to those in fresh arenas throughout the experimental time (Fig 4B). In small arenas, the velocities of CS males over 60 min were unaffected by pre-conditioning the arenas with male (Fig 4C) or female fly (Fig 4D).

The locomotion of mutant w1118 in fresh and pre-conditioned arenas was also examined. In large arenas, w1118 males traveled slowly at around 5.0 cm/min for 1 h. However, w1118 males traveled initially at 35.0 cm/min in male pre-conditioned arenas and maintained velocities at 20–35 cm/min for 1 h. The velocities in pre-conditioned arenas greatly increased compared with those in fresh arenas (*P* = 0.017, repeated measures ANOVA) (Fig 4E). In female pre-conditioned arenas, w1118 males remained inactive. The velocities were the same as those in fresh arenas (Fig 4F). In small arenas, there was no effect of male (Fig 4G) or female pre-conditioning (Fig 4H) on the fast locomotion of w1118 males.

In summary, in large arenas both CS and w1118 male flies increased locomotion in response to pre-conditioning by male but not female counterparts, indicating that male flies selectively increased locomotion in response to male pre-conditioning. In small arenas, CS and w1118 male flies maintained intense and persistent locomotion in fresh and pre-conditioned arenas at the same levels. There was no additional increase of locomotion with male pre-conditioning. Thus, both CS and w1118 males were highly sensitive to spatial restriction.

### Step size in arena with spatial restriction

**Fig 5:**
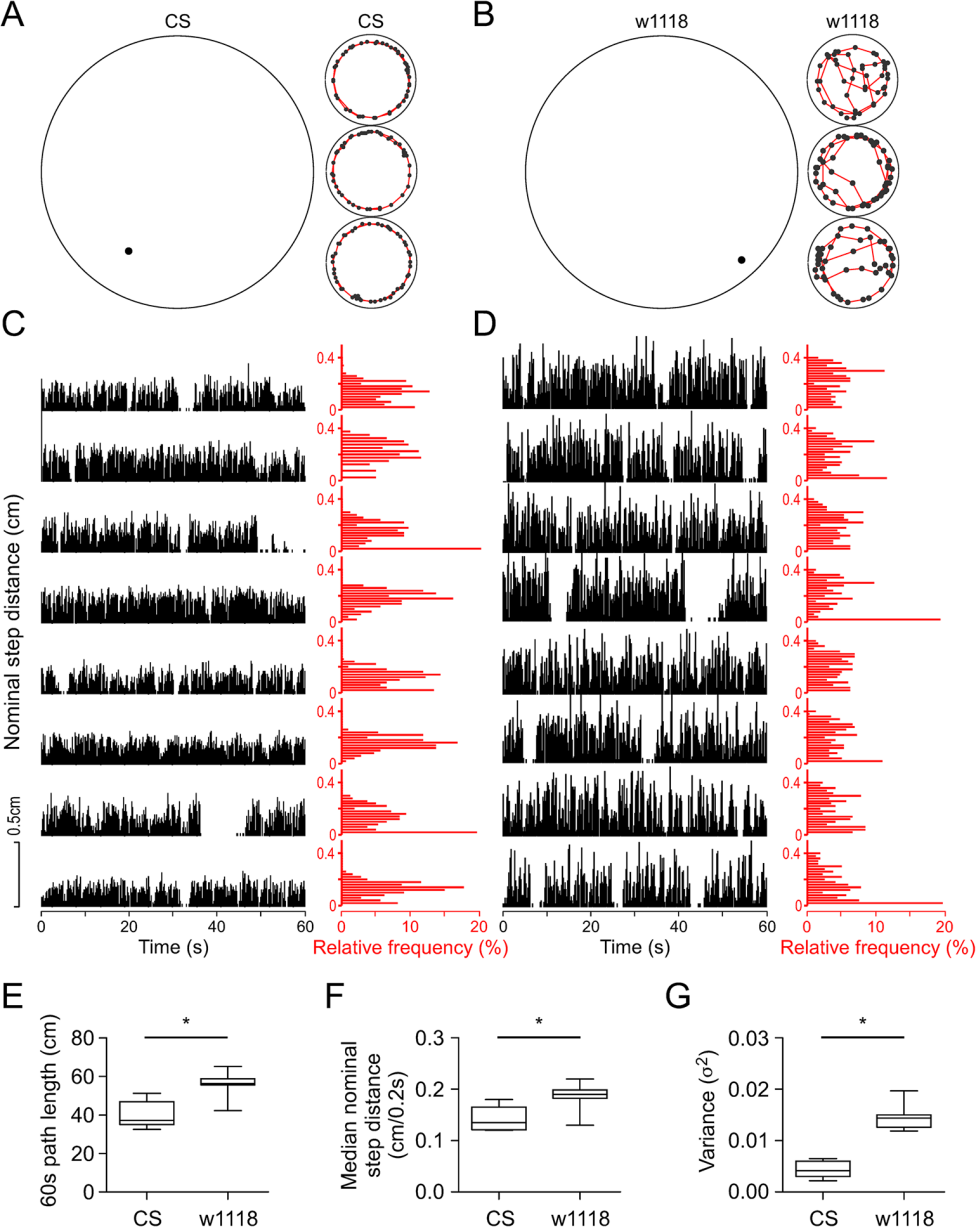
Step size with spatial restriction in CS and w1118 flies. (A, B) Diagrams illustrating relative locomotion of single CS or w1118 male flies in large or small arenas. The positions of individual fly were measured every 0.2s. In large arenas positional information is shown for 60s. For simplicity, only 10s of locomotion is shown in small arenas. (C) Nominal step distance (cm/0.2s) of 60s activity in CS male flies (n = 8). The histogram shows the relative frequency (%) of nominal step distance with bin width of 0.02 cm/0.2s. (D) Same as C for w1118 males (n = 8). (E) The 60s path length traveled in small arenas by CS (n = 8) and w1118 male flies (n = 8). (F) Median nominal step distance in small arenas in CS (n = 8) and w1118 male flies (n = 8). (G) Variance of nominal step distance within 60s for CS (n = 8) and w1118 flies (n = 8) in small arenas. The variance from each fly was calculated based on 300 data points of nominal step distance within 60s. The box plots were applied in E-G to indicate the median, interquartile range and whiskers (min and max). * denotes *P* < 0.05.

In large arenas, CS and w1118 males moved slowly at around 5–10 cm/min for 1 h. Many males displayed motionless periods of at least 60 s. In small arenas, however, CS and w1118 males traveled fast at around 40–50 cm/min and continued to walk almost non-stop (Fig 5A and 5B). In addition, CS males remained on the perimeter and traveled with a steady apparent step size (Fig 5A) while w1118 males traveled along the perimeter but also entered or crossed the open area with large variation in apparent step size (Fig 5B).

To evaluate the step size between CS and w1118 flies, we analyzed 60 s of locomotion by measuring the distance travelled in 0.2 s, which is equivalent to the average step period of a single leg at a body speed around 40–50 cm/min [40]. The distances per 0.2 s (the nominal step distance) in CS males were predominantly < 0.25 cm. The histogram plots (with bin width of 0.02 cm / 0.2 s) showed the most frequent nominal step distances in 60 s were less than 0.20 cm in most individuals (Fig 5C). In w1118 males, the nominal step distances were in a broad range of 0–0.5 cm. The histogram analysis showed that the most frequent nominal step distances were around or higher than 0.2 cm (Fig 5D). The 60 s path length in CS flies was shorter than that of w1118 flies (*P* < 0.05, Mann-Whitney test) (Fig 5E). The median nominal step distance in CS was shorter than that of w1118 (*P* < 0.05, Mann-Whitney test) (Fig 5F), and the median variance of nominal step distance in CS was smaller than that of w1118 (*P* < 0.05, Mann-Whitney test) (Fig 5G). Thus, although both CS and w1118 males walked continuously in small arenas, CS males walked with shorter 60 s path length, shorter nominal step distance and smaller variance than w1118 males.

### Boundary preference under spatial restriction

**Fig 6:**
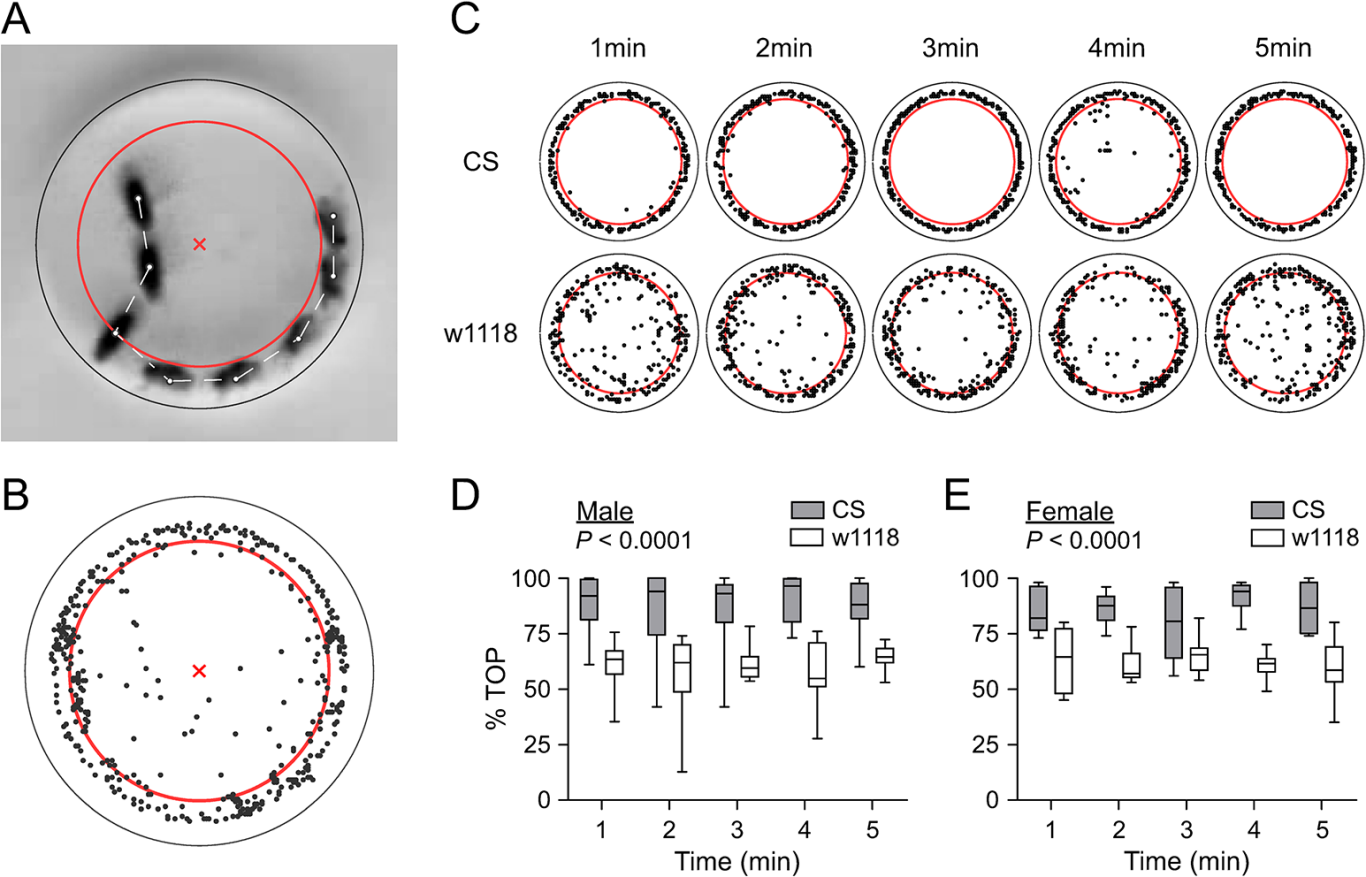
Boundary preference in small arenas in CS and w1118 flies. (A) Composite image of eight superimposed frames indicating the locations of single fly with 0.2s interval. The black outer circle indicates the edge of the arena. The red inner circle indicates a standard border delineating the perimeter zone. Black and red circles are spaced apart by half the length of the fly. White lines show the fly trajectory. Red cross (×) indicates the center of arena. If the center of the fly is on the red circle or within the area between two circles, the fly is deemed to be on the perimeter. See Materials and Methods for further description. (B) Illustration of fly centers (black dots) for 300 locations within 1min activity in a small arena. The standard border (red circle) and the center of arena (red cross) are indicated for reference. (C) Positions of a single CS and w1118 fly for 5 consecutive minutes in small arena. (D, E) %TOP during first five consecutive minutes for CS (n = 8) and w1118 (n = 8) male (D) or female (E) flies. The *P* values are the statistical results of repeated measures ANOVA comparing genotypes.

High boundary preference in circular arenas has been observed in wildtype CS flies [4, 22–24]. By overlapping 60 frames during a min of locomotion, we found that CS flies spent a high proportion of time on the perimeter of small arenas (see Fig 2). We quantified and compared the percentage of time on the perimeter (% TOP) within a minute between CS and w1118 flies. A circular line within the arena with a distance from the edge equivalent to half the size of the fly was used as a standard (Fig 6A, and supplementary information). A fly with its center of mass located on the standard or between the standard and the edge was judged as being on the perimeter. This criterion was highly conservative because the positions of flies that were able to physically contact the side wall were included to be on the perimeter in the following two typical situations: (1) when a fly was on the side wall, and (2) when a fly was on the boundary of top or bottom walls with its major body axis forming an acute angle to the side wall during locomotion. The criterion was also specific to each fly (see Methods) and thus should precisely determine the parameter %TOP.

The center of mass of a fly was calculated every 0.2 s. Positional information of individual flies within a time period was reconstructed as the distribution of the centers (represented as dots) in the circular arena (Fig 6B). We evaluated the positions of CS and w1118 flies within each of 5 consecutive minutes. CS male appeared in the perimeter zone for almost every time point within a minute, and retained a high preference for the perimeter from the first to the fifth minute. In contrast, w1118 male individuals traveled throughout the area of the arena within a minute, although the flies displayed a trend for boundary preference. There were many instances when w1118 flies were out of the perimeter zone (Fig 6C). The %TOP per minute in CS males was 88.5 ± 4.7% (n = 8, values here and following are present as Mean ± SE unless otherwise indicated) in the first minute and maintained at steady levels without decline for five consecutive minutes (repeated measures ANOVA). The %TOP per minute in w1118 males was 60.6 ± 4.2% (n = 8) in the first minute and remained comparable without decline for five consecutive minutes (repeated measures ANOVA). The %TOPs in CS males were clearly higher than those in w1118 males (*P* < 0.0001, repeated measures ANOVA) (Fig 6D). In female flies, %TOP per minute was 85.0 ± 3.5% in CS (n = 8) and 62.4 ± 5.0% in w1118 (n = 8) in the first minute. The %TOPs remained consistent within strain for five consecutive minutes (repeated measures ANOVA). The %TOPs in CS females were higher than those in w1118 females (*P* < 0.0001, repeated measures ANOVA) (Fig 6E). Therefore, CS flies displayed higher boundary preference than w1118 in arenas with spatial restriction, and the difference was observed in both male and female flies.

### Independence between the w gene and boundary preference in wildtype

The w1118 strain contains isogenic X, second and third chromosomes and a null mutant allele *w* ^1118^ on the X chromosome [30, 31]. We addressed whether the boundary preference in small arenas can be attributed to: (1) the *w* gene, (2) the genetic background excluding *w* locus, or (3) the cytoplasmic background in wildtype fly.

**Fig 7:**
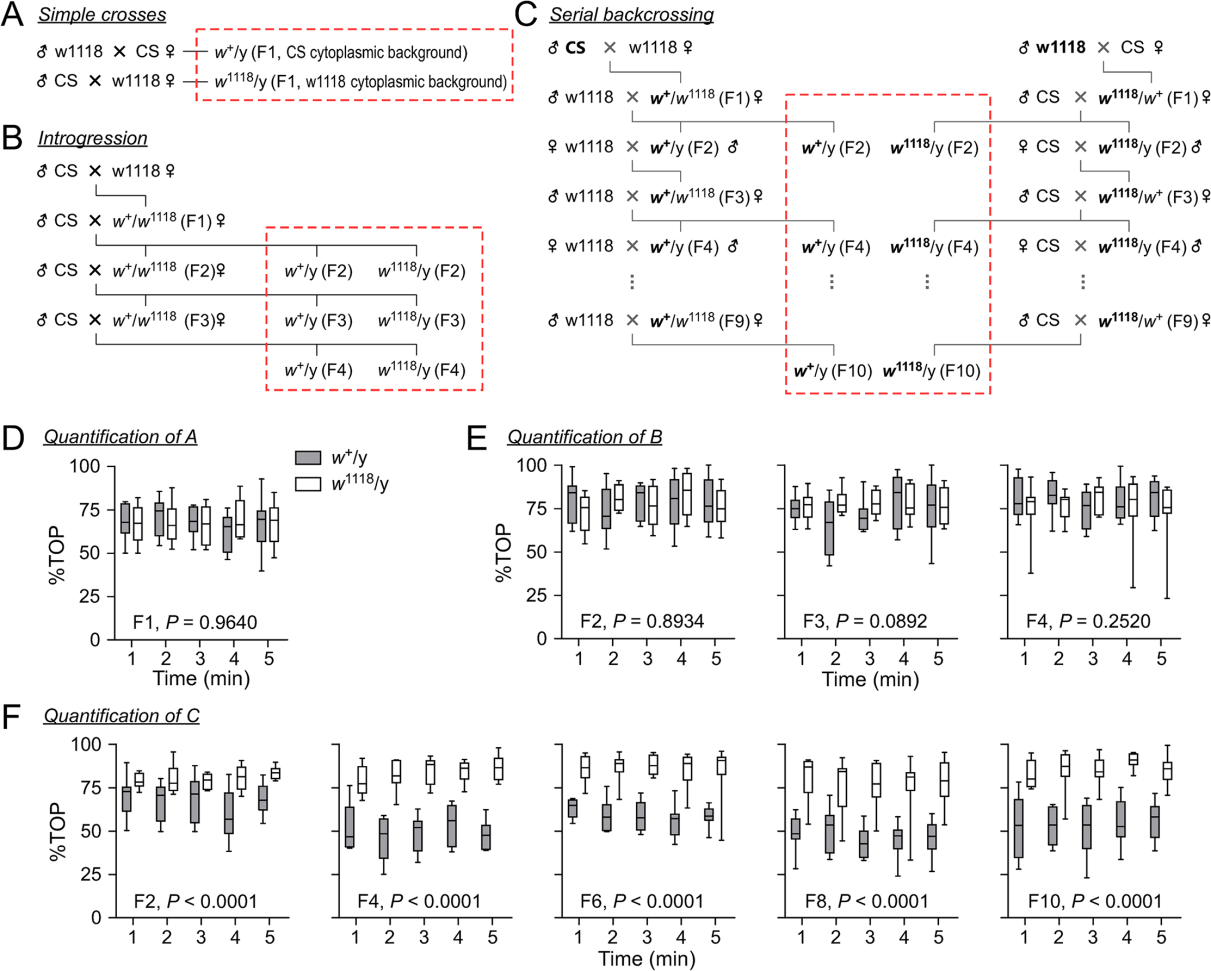
The analysis of genetic factors associated with boundary preference in wildtype. (A, B, C) The scheme of fly crosses to generate flies with desired genetic contents and cytoplasmic background. The simple crosses, introgression and serial backcrossing are illustrated. The progeny used for testing are indicated in the red dashed rectangles. (D) Quantification of %TOP for male flies generated from the simple cross A. Flies carrying *w* ^+^ (n = 8, grey boxes) and *w* ^1118^ (n = 8, open boxes) were tested. (E) Quantification of %TOP for male flies generated from introgression B. Flies carrying *w* ^+^ (n = 8, grey boxes) and *w* ^1118^ (n = 8, open boxes) in the same generation were tested. (F) Quantification of %TOP for male flies generated by serial backcrossing C. Flies carrying *w* ^+^ allele in w1118 genetic background (n = 8, grey boxes) and *w* ^1118^ allele in CS genetic background (n = 8, open boxes) in the same generation were tested. The *P* values are from repeated measures ANOVA comparing genotypes.

Three types of crosses were performed between CS and w1118 to exchange the *w* alleles or the genetic or cytoplasmic background. First, a simple cross between male w1118 and female CS, and the reciprocal cross between male CS and female w1118 were conducted. The simple crosses yielded two different F1 male progenies: (1) *w* ^+^/y (F1), which carried wildtype *w* allele (*w* ^+^) on the X chromosome from CS flies, a half genetic background of second and third chromosomes from CS and a half from w1118, and CS cytoplasmic background; (2) *w* ^1118^/y (F1), which carried mutant *w* allele (*w* ^1118^) on the X chromosome from w1118 flies, a half genetic background of second and third chromosomes from CS and a half from w1118, and w1118 cytoplasmic background (Fig 7A). Second, introgression was conducted to gradually replace the genetic content of w1118 with the counterpart of CS flies [34]. Male CS was initially crossed with female w1118, and the heterozygous F1 female progeny was backcrossed with CS male again. From F2 to F3, the female progeny were continuously backcrossed with male CS. The F2—F4 male progenies were separated into two groups of flies carrying either *w* ^+^ or *w* ^1118^ allele in each group. These two groups of males in the same generation contained different *w* alleles and synchronized genetic background and identical cytoplasmic background from the same parents. Through introgression, the genetic contents from F2 to F4 males were gradually shifted towards wildtype (Fig 7B). Third, serial backcrossing was performed to exchange *w* alleles between CS and w1118 flies. The male ancestor of CS or w1118 was crossed into female w1118 or CS to produce *w* ^+^/*w* ^1118^ heterozygous female progeny. These progeny were then backcrossed with w1118 or CS stock. By selecting *w* ^+^ or *w* ^1118^-carrying progeny and backcrossing with w1118 or CS stock for nine generations, the *w* locus remained while the genetic background from the male ancestor was gradually diluted. *w* ^+^ or *w* ^1118^-carrying male flies in F2, F4, F6, F8 and F10 were collected and tested. Theoretically, from F2 to F10, *w* ^+^-carrying males contained w1118 genetic content with increasing probability from 75% to 99.9% for the second or third chromosome, whereas *w* ^1118^-carrying males contained CS genetic content with increasing probability from 75% to 99.9% for the second or third chromosome. In addition, the genetic content on the X chromosome, excluding the *w* locus, had a high probability of being exchanged in the *w* ^+^/*w* ^1118^ heterozygous females through cross-over (Fig 7C).

We found that in male flies from the simple crosses, the %TOP per minute was 67.9 ± 3.7% in *w* ^+^/y flies (n = 8), and 66.7 ± 3.8% in *w* ^1118^/y flies (n = 8) in the first minute. The %TOP remained the same over time within the strain in five consecutive minutes. There was no difference of %TOP between *w* ^+^/y and *w* ^1118^/y flies from the simple crosses (*P* = 0.9640, repeated measures ANOVA) (Fig 7D). Thus, the *w* ^+^ allele, the cytoplasmic background from CS, or the combination of *w* ^+^ allele and the CS cytoplasmic background did not confer higher boundary preference in *w* ^+^/y flies. These findings were supported by the tests in F2-F4 males from the introgression. There was no difference of %TOP between *w* ^+^/y and *w* ^1118^/y flies in F2 (*P* = 0.8934, repeated measures ANOVA), F3 (*P* = 0.0892, repeated measures ANOVA), or F4 (*P* = 0.2520, repeated measures ANOVA) (Fig 7E). Therefore, again, the *w* ^+^ allele did not confer a higher boundary preference in *w* ^+^/y flies compared with *w* ^1118^/y flies. Results confirmed that the genetic background, excluding the *w* ^+^ allele, contributed to higher boundary preference in wildtype. These conclusions were further confirmed by the tests in male flies from serial backcrossing. From F2 to F10, the %TOP in *w* ^+^/y flies was lower than that in *w* ^1118^/y flies in F2 (*P* < 0.0001, repeated measures ANOVA), F4 (*P* < 0.0001, repeated measures ANOVA), F6 (*P* < 0.0001, repeated measures ANOVA), F8 (*P* < 0.0001, repeated measures ANOVA), and F10 (*P* < 0.0001, repeated measures ANOVA) (Fig 7F). Thus, the genetic background other than *w* ^+^ allele in wildtype was associated with high boundary preference. The *w* ^+^ allele was clearly not associated with higher boundary preference in wildtype fly.

**Fig 8:**
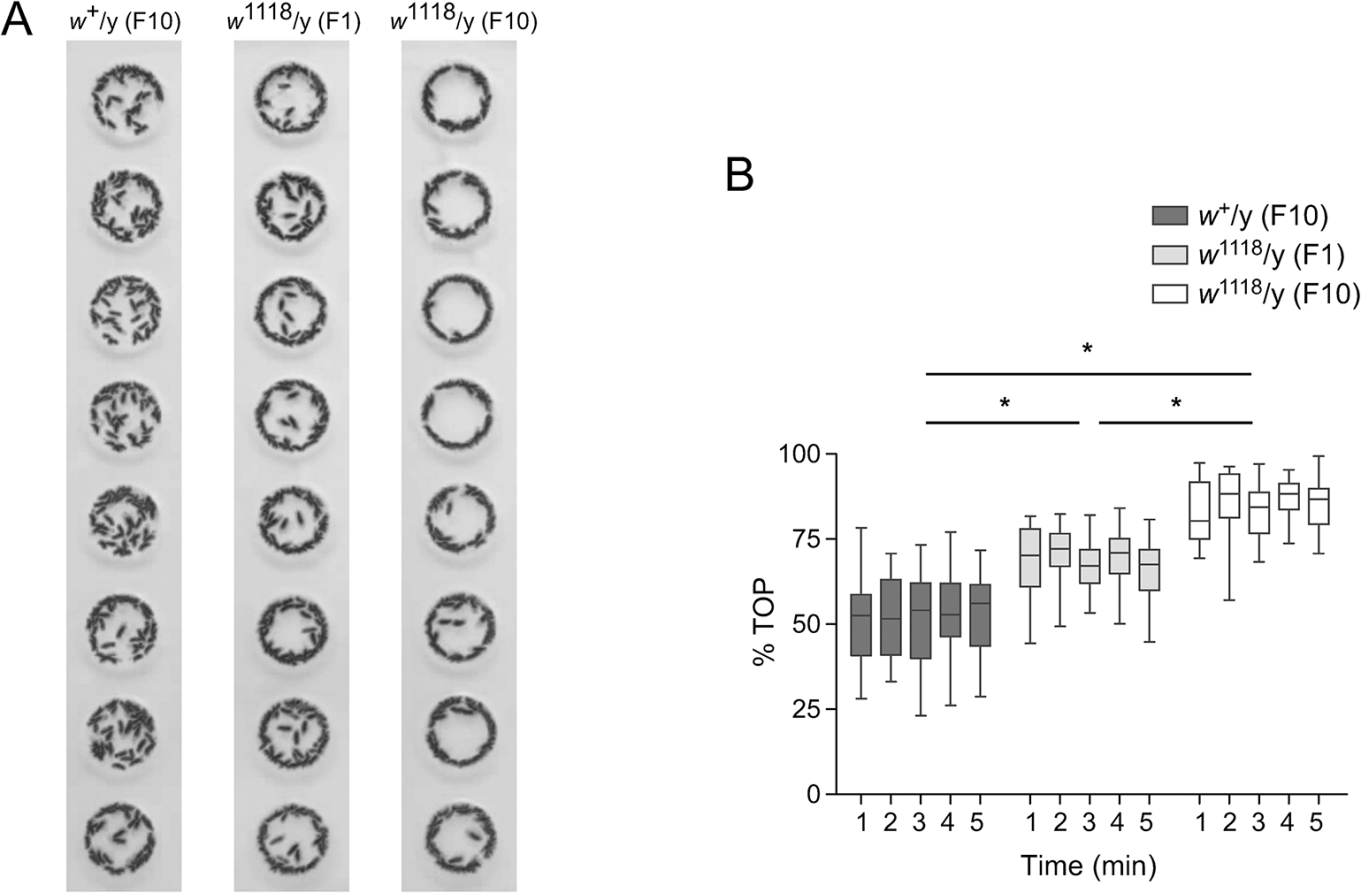
Correlation between wildtype genetic background and boundary preference. (A) Composite images of 60 superimposed frames from video of 1min activity. Each arena contains one fly. Flies carrying varying amount of wildtype genetic background (n = 8 for each genotype) were tested. See text for further description. (B) The box plots of %TOP for flies with different amount of wildtype genetic background during five consecutive minutes. * denotes *P* <0.05 with repeated measures ANOVA and Bonferroni's multiple comparison.

To examine the correlation between the content of wildtype genetic background and boundary preference in small arenas, the %TOP was evaluated in three different males: (1) *w* ^+^/y (F10), which carried the least amount of wildtype genetic content; (2) *w* ^1118^/y (F1), which carried half the wildtype genetic content; and (3) *w* ^1118^/y (F10), which carried the most wildtype genetic content. *w* ^+^/y (F10) flies displayed a high probability of being located in the open area of arenas; *w* ^1118^/y (F10) flies had a high probability of moving on the perimeter; and *w* ^1118^/y (F1) flies were intermediate between *w* ^+^/y (F10) and *w* ^1118^/y (F10) flies (Fig 8A). There was a significant difference of %TOP between *w* ^+^/y (F10) and *w* ^1118^/y (F1) flies (*P* < 0.05, repeated measures ANOVA with Bonferroni post-test), between *w* ^1118^/y (F1) and *w* ^1118^/y (F10) flies (*P* < 0.05, repeated measures ANOVA with Bonferroni post-test), and between *w* ^+^/y (F10) and *w* ^1118^/y (F10) flies (*P* < 0.05, repeated measures ANOVA with Bonferroni post-test) (Fig 8B). Thus, the %TOP was highest in *w* ^1118^/y (F10) flies, lowest in *w* ^+^/y (F10) flies and in the middle levels in *w* ^1118^/y (F1) flies. Additionally, there was a significant linear trend of %TOP among *w* ^+^/y (F10), *w* ^1118^/y (F1) and *w* ^1118^/y (F10) flies (*P* < 0.0001, R^2^ = 0.9886, repeated measures ANOVA with posthoc for linear trend). Therefore, the %TOP was tightly correlated with the content of genetic background in wildtype and not the *w* gene.

## Discussion

The locomotion of *Drosophila* adults display features related to the size of the arena [1, 2, 5, 22, 24, 25], however, the effect of spatial restriction on locomotion has not previously been investigated in detail. In this study we established a protocol and examined the characteristics of locomotion with spatial restriction in adult flies.

Our major finding is that male flies increase locomotion under spatial restriction. The space increase did not result in longer path lengths per unit time. Instead, flies were motionless for long periods, during which behaviors associated with resting, such as grooming of antennae and wings, were common. With spatial restriction the motionless periods were not obvious or greatly shortened, and flies walked continuously throughout the duration of the experiment with a steady velocity. Spatial restriction thus initiates selective locomotor activity related to arena size. The transition from mostly inactive to highly active states was observed in different genetic strains, suggesting that it is a common feature related to arena size. These findings are consistent with a previous report that relentless activity is observed in a small rectangular arena [2].

Selective locomotion was also observed in response to arena pre-conditioning. Specifically, male but not female pre-conditioning increased locomotion of male flies, suggesting the male-male competition for resources such as territory or female mates. The pre-conditioning effect by male fly is common to wildtype and the mutant w1118. A broad odorant receptor gene *Or83b* is proposed to mediate behavioral and electrophysiological responses to many odorants [41]. Both wildtype and w1118 mutant carry normal *Or83b* allele, consistent with their similar responses to arena pre-conditioning assuming this is mediated by olfactory information. Future experiments using this anosmic mutant will help to determine the sensory basis for the preconditioning effect. Spatial restriction, however, abolished or overwhelmed the pre-conditioning effects. This finding suggests that spatial restriction is the predominant environmental stimulus triggering increased locomotion. The intensity of locomotion may have been maximal in the restricted situation, without the ability to increase velocity in response to the combination of spatial restriction and arena pre-conditioning.

Male and female flies displayed dimorphism in their locomotor responses to spatial restriction. Wildtype female flies moved faster in larger arenas. Unlike wildtype male flies, female flies walked continuously with few stops in large arenas. On a flat horizontal surface without adjacent barrier the average speed of a free-walking female fly is 148 cm/min (28 mm/s) [40], which is even higher than the velocity we measured in large circular arenas. Thus, female flies possess the ability to walk fast if the arena space is permissive. Our findings indicate that wildtype male and female flies use different locomotor strategies in large arenas. Males tended to rest in the open area or on the edge, whereas females tended to walk continuously in large arenas. Female w1118 flies in large and small arenas had similar locomotion, which was different from male w1118 flies for which walking speed markedly increased in small arenas compared with large arenas. Therefore, both wildtype and mutant flies display dimorphic characteristics of locomotion in response to spatial restriction.

Male flies display strain-specific features in locomotion with spatial restriction. Wildtype males walked slower and steadier than mutant flies in small arenas. Individual flies of the same strain are highly consistent in the step size, suggesting that locomotion is intrinsically different between strains. In addition, wildtype flies showed a strong preference for walking on the periphery, constrained into circular paths, and this is different from mutant flies whose trajectories were irregularly shaped. The head of mutant w1118 contains reduced quantities of serotonin and dopamine [42, 43], which are neurotransmitters closely associated with locomotor performance and memory [43–47]. Alterations of biogenic amines could thus be related to the irregular shapes of locomotor trajectory in the mutant fly.

Males tended to continue walking without stopping. Even in the mutant strain, males tended to move quickly to cross the open area to reach another location of the edge, although there was a higher probability of being away from the perimeter zone and stopping. During locomotion males walked on the side wall, or in the perimeter zone of the bottom or top walls. In either situation the males faced towards the arena edge but not the central open region. These findings suggest that male flies perform active exploration in the small arenas. This exploratory behavior could represent an innate response to environmental stimulus such as confinement. With spatial restriction, flies might initiate exploration to search for an exit. Slow walking in the rim zone of an arena has been suggested as being associated with exploratory behavior [24].

Wildtype flies display a boundary preference in circular or square arenas [1, 4, 22–25]. We show here a consistent preference for the perimeter during intense and persistent locomotion in small arenas. Boundary preference was observed in both wildtype and mutant flies though it was more obvious in wildtype. The mutant strain carries the *w* ^1118^ allele on the X chromosome [30, 31]. Genetic analysis including simple crosses, introgression and serial backcrossing together revealed that the *w* gene was not associated with boundary preference in wildtype flies. Rather, the genetic background on the X, second or third chromosomes in wildtype was tightly associated with a high degree of boundary preference in small arenas. The boundary preference in wildtype flies requires intact visual function [4, 22]. However, we show that the *w* ^1118^ allele, and presumably any related visual impairment, is independent of the phenotype of boundary preference, because by generating a fly line containing wildtype genetic background, with the presence of *w* ^1118^ allele, the high boundary preference was retained. It is possible that some of these behaviours, or the difference between CS and w1118, were mediated visually. We believe this is unlikely because the results were not different in experiments performed under dim red illumination (Xiao and Robertson unpublished observations). Furthermore, the level of wildtype genetic background determined the extent of boundary preference. Therefore, our findings refine the association between boundary preference and visual function, and highlight that the *w* gene and its associated visual function is independent of boundary preference in wildtype fly.

## Conclusions

*Drosophila* adults had sexually dimorphic locomotor strategies in our experiments but it was clear that, independently of sex, confinement in a small arena promoted continuous rapid locomotion largely around the perimeter that lasted for the duration of the assay (>1 h). This had the appearance of a stereotypic behavioral response to claustrophobic conditions, suggesting exploration in search of an exit. The strain-specific features in locomotion with confinement indicated a role for genetic determination. The independence of the *w* gene from high boundary preference in wildtype fly demonstrated that any *w*- related visual impairment was not responsible for the altered phenotype of boundary preference in the mutant fly. We also found that, in a large arena where they would normally be inactive, males could be stimulated to similar continuous rapid locomotion if the arena had been pre-conditioned by prior occupation with a male. The fact that this was sex-specific, and female preconditioning did not have the same effect, suggests that this locomotor strategy was associated with competitive behavior between males. Thus it may be possible to separately assay locomotor activity dependent on distinct motivational states and our protocol will facilitate the genetic dissection of the underlying neural control pathways.

## Supporting Information

S1 Text Supporting experimental procedures. (DOCX)

S1 Appendix Contains the following files: Code for background learning (BgLearning.cpp); Code for fly tracking in 3 × 3 arenas (FlyTrack9.cpp); Code for fly size estimation in 8 × 8 arenas (FlySize64.cpp); Code for fly tracking in 8 × 8 arenas (FlyTrack64.cpp). (ZIP)
