# Supplementary material for: Locomotion Induced by Spatial Restriction in Adult Drosophila
Source: PLoS One. 2015 Sep 9;10(9):e0135825. doi: 10.1371/journal.pone.0135825 (PMC4564261; doi:10.1371/journal.pone.0135825)
Supplement: S1 Text — (DOCX) [file pone.0135825.s001.docx]

**S1. Supporting experimental procedures**

We used a Logitech webcam C905 and its associated software for video capture. Based on the format of captured videos we developed specialized scripting language using open source software for locomotor assays.

The Logitech C905 webcam had several advantages: (1) frame resolution could reach 1600 × 1200 pixels; (2) frame rate could reach 15 frames per second (fps), which is good enough for the locomotor assays; (3) the camera could capture images for several hours with stable performance; (4) the camera distortion rate measured at 0.7%; (5) the color and sound backgrounds could be suppressed to reduce video size. A typical 1h video of 1600 × 1200 pixels at 15fps is around 300 Megabytes, which is reasonably small in file size yet containing sufficient information for large scale analysis of locomotion.

There are a few fly tracking software packages either commercially or freely available [1-5]. However, using developed software for a specific behavioral project may be unsatisfactory because of factors such as customized experimental settings, specialized video format, preferred parameters for analysis, and the necessity of special computer platform or running environments. Thus we used custom-written code to compute the desired parameters using open source scripting language. With the help of Microsoft Visual C++ 2008 Express (free version) and Open Source Computer Vision 2.0 (OpenCV2.0) (<http://opencv.org/>), coding for fly tracking becomes a routine procedure suitable for desired experimental settings. For this study, we developed a series of codes each of which can implement a single task such as calculating center of mass and estimating fly size. These codes are small (less than 40K) yet powerful and sufficient to calculate the parameters (i.e. position information of individual flies). The goal was to detect the center of mass of each object in each frame after which a number of parameters (such as path length, off-center distance and walking speed) could be calculated. The major procedures included, (1) learning a specific background from multiple frames of a video, (2) comparing the difference between each frame and background, (3) computing the center of mass of each object, and (4) calculating the parameters (e.g. distance moved, and distance during a specific period). The arena depth was 3mm, which was high enough for the fly to turn around but prevents the fly from crawling vertically, thus the two-dimensional calculated path length maximally reflects the distance travelled. Calibration was performed by placing a ruler in the viewing frame for pixel-mm conversion.

To avoid the wobble effect of camera, the positional information was evaluated five times per second with an interval of 0.2s. The time interval of 0.2s was equivalent to the duration of one step of a single leg at the walking speed of 40-50 cm/min [6]. The position calculation with 0.2s interval has been successfully applied in a previous study [7].

Fly size was estimated by calculating the area of fly body. A total of 100 frames from the first 20s locomotion were extracted with 0.2s interval. After subtracting the background the blob area of each object was smoothed by a Gaussian 3 × 3 filter and the square pixels for the area were calculated. The middle 50% of the 100 estimations was used as representation of fly area. We chose middle 50% because of the following considerations: (1) the maximum value could overestimate the fly size by having both fly body and shadow involved, and (2) the lower 25% might contain the values estimated from front views of a fly. The evaluated fly area was in a shape of oval-like. We calculated the ratio of major/minor axis of 5-day old w1118 flies and obtained a ratio of 2.1 for both male and female flies. Thus the ratio of 2.1 was applied for calculating the major axis of a fly by using the ellipse equation: area = πab, where *a* and *b* are the lengths of semi-major and semi-minor axis. The calibration was performed by comparing the estimations to the measurements of major axis in the image. The percent error was less than 10%.

The codes for background computing, fly tracking and fly size estimation were provided in the appendix (S2 Appendix).

**References**

1. Branson K, Robie AA, Bender J, Perona P, Dickinson MH. High-throughput ethomics in large groups of Drosophila. Nat Methods. 2009;6(6):451-7. doi: 10.1038/nmeth.1328. PubMed PMID: 19412169; PubMed Central PMCID: PMCPMC2734963.

2. Colomb J, Reiter L, Blaszkiewicz J, Wessnitzer J, Brembs B. Open source tracking and analysis of adult Drosophila locomotion in Buridan's paradigm with and without visual targets. PLoS One. 2012;7(8):e42247. doi: 10.1371/journal.pone.0042247. PubMed PMID: 22912692; PubMed Central PMCID: PMCPMC3415391.

3. Donelson NC, Donelson N, Kim EZ, Slawson JB, Vecsey CG, Huber R, et al. High-resolution positional tracking for long-term analysis of Drosophila sleep and locomotion using the "tracker" program. PLoS One. 2012;7(5):e37250. doi: 10.1371/journal.pone.0037250. PubMed PMID: 22615954; PubMed Central PMCID: PMCPMC3352887.

4. Kabra M, Robie AA, Rivera-Alba M, Branson S, Branson K. JAABA: interactive machine learning for automatic annotation of animal behavior. Nat Methods. 2013;10(1):64-7. doi: 10.1038/nmeth.2281. PubMed PMID: 23202433.

5. Dankert H, Wang L, Hoopfer ED, Anderson DJ, Perona P. Automated monitoring and analysis of social behavior in Drosophila. Nat Methods. 2009;6(4):297-303. doi: 10.1038/nmeth.1310. PubMed PMID: 19270697; PubMed Central PMCID: PMCPMC2679418.

6. Mendes CS, Bartos I, Akay T, Márka S, Mann RS. Quantification of gait parameters in freely walking wild type and sensory deprived Drosophila melanogaster. Elife. 2013;2:e00231. doi: 10.7554/eLife.00231. PubMed PMID: 23326642; PubMed Central PMCID: PMCPMC3545443.

7. Valente D, Golani I, Mitra PP. Analysis of the trajectory of Drosophila melanogaster in a circular open field arena. PLoS One. 2007;2(10):e1083. doi: 10.1371/journal.pone.0001083. PubMed PMID: 17957265; PubMed Central PMCID: PMCPMC2031922.
