# Supplementary material for: Locomotion Induced by Spatial Restriction in Adult Drosophila
Source: PLoS One. 2015 Sep 9;10(9):e0135825. doi: 10.1371/journal.pone.0135825 (PMC4564261; doi:10.1371/journal.pone.0135825)
Supplement: S1 Appendix — (ZIP) [file pone.0135825.s002.zip › FlySize64.pdf]

```cpp
/***********************************************
FlySize64.cpp
***********************************************/
#include <stdafx.h>
#include <iostream>
#include <fstream>
#include <sstream>
#include <string>
#include <cv.h>
#include <highgui.h>
#include <cxcore.h>
using namespace std;

int main(int argc, char** argv)
{
    IplImage*  pframe = NULL;
    IplImage*  frame = NULL;
    IplImage*  background = NULL;
    IplImage*  flytrack = NULL;

    CvCapture* capture = NULL;

    int        start_frame = 0;
    int        total_frames = 0;
    int        analysis_step = 0;
    int        tail_length = 0;

    int        TL_ROIx = 0, TL_ROIy = 0;
    int        BR_ROIx = 0, BR_ROIy = 0;

    int        Top_0x = 0;
    int        Top_1x = 0;
    int        Top_2x = 0;
    int        Top_3x = 0;
    int        Top_4x = 0;
    int        Top_5x = 0;
    int        Top_6x = 0;
    int        Top_7x = 0;
    int        Top_8x = 0;

    int        Left_0y = 0;
    int        Left_1y = 0;
    int        Left_2y = 0;
    int        Left_3y = 0;
    int        Left_4y = 0;
    int        Left_5y = 0;
    int        Left_6y = 0;

    int        Left_7y = 0;
    int        Left_8y = 0;

    int        fly01x = 0, fly01y = 0;
    int        fly02x = 0, fly02y = 0;
    int        fly03x = 0, fly03y = 0;
    int        fly04x = 0, fly04y = 0;
    int        fly05x = 0, fly05y = 0;
    int        fly06x = 0, fly06y = 0;
    int        fly07x = 0, fly07y = 0;
    int        fly08x = 0, fly08y = 0;
    int        fly09x = 0, fly09y = 0;
    int        fly10x = 0, fly10y = 0;
    int        fly11x = 0, fly11y = 0;
    int        fly12x = 0, fly12y = 0;
    int        fly13x = 0, fly13y = 0;
    int        fly14x = 0, fly14y = 0;
    int        fly15x = 0, fly15y = 0;
    int        fly16x = 0, fly16y = 0;
    int        fly17x = 0, fly17y = 0;
```

```
int        fly18x = 0, fly18y = 0;
int        fly19x = 0, fly19y = 0;
int        fly20x = 0, fly20y = 0;
int        fly21x = 0, fly21y = 0;
int        fly22x = 0, fly22y = 0;
int        fly23x = 0, fly23y = 0;
int        fly24x = 0, fly24y = 0;
int        fly25x = 0, fly25y = 0;
int        fly26x = 0, fly26y = 0;
int        fly27x = 0, fly27y = 0;
int        fly28x = 0, fly28y = 0;
int        fly29x = 0, fly29y = 0;
int        fly30x = 0, fly30y = 0;
int        fly31x = 0, fly31y = 0;
int        fly32x = 0, fly32y = 0;
int        fly33x = 0, fly33y = 0;
int        fly34x = 0, fly34y = 0;
int        fly35x = 0, fly35y = 0;
int        fly36x = 0, fly36y = 0;
int        fly37x = 0, fly37y = 0;
int        fly38x = 0, fly38y = 0;
int        fly39x = 0, fly39y = 0;
int        fly40x = 0, fly40y = 0;
int        fly41x = 0, fly41y = 0;
int        fly42x = 0, fly42y = 0;
int        fly43x = 0, fly43y = 0;
int        fly44x = 0, fly44y = 0;
int        fly45x = 0, fly45y = 0;
int        fly46x = 0, fly46y = 0;
int        fly47x = 0, fly47y = 0;
int        fly48x = 0, fly48y = 0;

int        fly49x = 0, fly49y = 0;
int        fly50x = 0, fly50y = 0;
int        fly51x = 0, fly51y = 0;
int        fly52x = 0, fly52y = 0;
int        fly53x = 0, fly53y = 0;
int        fly54x = 0, fly54y = 0;
int        fly55x = 0, fly55y = 0;
int        fly56x = 0, fly56y = 0;
int        fly57x = 0, fly57y = 0;
int        fly58x = 0, fly58y = 0;
int        fly59x = 0, fly59y = 0;
int        fly60x = 0, fly60y = 0;
int        fly61x = 0, fly61y = 0;
int        fly62x = 0, fly62y = 0;
int        fly63x = 0, fly63y = 0;
int        fly64x = 0, fly64y = 0;

double        fly01area = 0.0;
double        fly02area = 0.0;
double        fly03area = 0.0;
double        fly04area = 0.0;
double        fly05area = 0.0;
double        fly06area = 0.0;
double        fly07area = 0.0;
double        fly08area = 0.0;
double        fly09area = 0.0;
double        fly10area = 0.0;
double        fly11area = 0.0;
double        fly12area = 0.0;
double        fly13area = 0.0;
double        fly14area = 0.0;
double        fly15area = 0.0;
double        fly16area = 0.0;
double        fly17area = 0.0;
double        fly18area = 0.0;
double        fly19area = 0.0;
```

```
double       fly20area = 0.0;
double       fly21area = 0.0;
double       fly22area = 0.0;
double       fly23area = 0.0;
double       fly24area = 0.0;
double       fly25area = 0.0;
double       fly26area = 0.0;
double       fly27area = 0.0;
double       fly28area = 0.0;
double       fly29area = 0.0;
double       fly30area = 0.0;
double       fly31area = 0.0;
double       fly32area = 0.0;
double       fly33area = 0.0;
double       fly34area = 0.0;
double       fly35area = 0.0;
double       fly36area = 0.0;
double       fly37area = 0.0;
double       fly38area = 0.0;
double       fly39area = 0.0;
double       fly40area = 0.0;
double       fly41area = 0.0;
double       fly42area = 0.0;
double       fly43area = 0.0;
double       fly44area = 0.0;
double       fly45area = 0.0;
double       fly46area = 0.0;
double       fly47area = 0.0;
double       fly48area = 0.0;

double       fly49area = 0.0;
double       fly50area = 0.0;
double       fly51area = 0.0;
double       fly52area = 0.0;
double       fly53area = 0.0;
double       fly54area = 0.0;
double       fly55area = 0.0;
double       fly56area = 0.0;
double       fly57area = 0.0;
double       fly58area = 0.0;
double       fly59area = 0.0;
double       fly60area = 0.0;
double       fly61area = 0.0;
double       fly62area = 0.0;
double       fly63area = 0.0;
double       fly64area = 0.0;


int        counter = 0;

if (argc ==2 && (capture = cvCreateFileCapture(argv[1])) != 0) {

    cout << "Analysis starting at frame (i.e. 1): ";
    cin >> start_frame;

    cout << "Total frames for analysis (i.e. 9000): ";
    cin >> total_frames;

    cout << "Analysis Step (1-15): ";
    cin >> analysis_step;

    cout << "Tail Length (1-10): ";
    cin >> tail_length;

    cout << "Enter ROI Top Left x Axis Value (in pixel): ";
    cin >> TL_ROIx;

    cout << "Enter ROI Top Left y Axis Value (in pixel): ";
```

```
cin >> TL_ROIy;

cout << "Enter ROI Bottom Right x Axis Value (in pixel): ";
cin >> BR_ROIx;

cout << "Enter ROI Bottom Right y Axis Value (in pixel): ";
cin >> BR_ROIy;

Top_0x = 10;
Left_0y = 10;
Top_8x = abs(BR_ROIx - TL_ROIx) - Top_0x;
Left_8y = abs(BR_ROIy - TL_ROIy) - Left_0y;

Top_1x = (int)(Top_0x + (Top_8x - Top_0x)/8);
Top_2x = (int)(Top_0x + 2*(Top_8x - Top_0x)/8);
Top_3x = (int)(Top_0x + 3*(Top_8x - Top_0x)/8);
Top_4x = (int)(Top_0x + 4*(Top_8x - Top_0x)/8);
Top_5x = (int)(Top_0x + 5*(Top_8x - Top_0x)/8);
Top_6x = (int)(Top_0x + 6*(Top_8x - Top_0x)/8);
Top_7x = (int)(Top_0x + 7*(Top_8x - Top_0x)/8);

Left_1y = (int)(Left_0y + (Left_8y - Left_0y)/8);
Left_2y = (int)(Left_0y + 2*(Left_8y - Left_0y)/8);
Left_3y = (int)(Left_0y + 3*(Left_8y - Left_0y)/8);
Left_4y = (int)(Left_0y + 4*(Left_8y - Left_0y)/8);
Left_5y = (int)(Left_0y + 5*(Left_8y - Left_0y)/8);
Left_6y = (int)(Left_0y + 6*(Left_8y - Left_0y)/8);
Left_7y = (int)(Left_0y + 7*(Left_8y - Left_0y)/8);

background = cvLoadImage("Background.jpg", 0);
cvSetImageROI(background, cvRect(TL_ROIx, TL_ROIy, abs(BR_ROIx - TL_ROIx), abs(BR_ROIy - TL_ROIy)));

flytrack = cvCloneImage(background);
frame = cvCloneImage(background);
cvZero(flytrack);
cvZero(frame);

cvNamedWindow("FlyTrack",1);

CvMemStorage* cstorage = NULL;
CvSeq*        contours = NULL;

CvMemStorage* pstorage = NULL;
pstorage = cvCreateMemStorage(0);

CvSeqWriter   writer;
CvSeq*        psequence = NULL;

cvStartWriteSeq(CV_32SC2, sizeof(CvSeq), sizeof(CvPoint), pstorage, &writer);
psequence = cvEndWriteSeq(&writer);

cvStartAppendToSeq(psequence, &writer);

CvMoments moments;
double M00, M01, M10;
CvPoint centers;
CvMat mat;

CvFont font;
cvInitFont(&font, CV_FONT_HERSHEY_PLAIN, 1, 2, 0, 1, 8);

ofstream myfile;
myfile.open("FlySize.txt");

for (int i = 0; i < start_frame; i++)
{
    pframe = cvQueryFrame(capture);
}
```

```
for( int i = start_frame; i <= start_frame + total_frames; i++) {

    pframe = cvQueryFrame(capture);
    counter ++;

    if (counter == analysis_step) {

        cvSetImageROI(pframe, cvRect(TL_ROIx, TL_ROIy, abs(BR_ROIx - TL_ROIx), abs(BR_ROIy - TL_ROIy)));

        cvCvtColor(pframe, frame, CV_BGR2GRAY);

        cvAbsDiff(frame, background, flytrack);
        cvThreshold(flytrack, flytrack, 40, 255, CV_THRESH_BINARY);

        cvErode(flytrack, flytrack, 0, 1);
        cvDilate(flytrack, flytrack, 0, 1);

        cvSmooth(flytrack, flytrack, CV_MEDIAN, 3, 3);

        cvRectangle(pframe, cvPoint(Top_0x, Left_0y), cvPoint(Top_8x,Left_8y), cvScalar(0, 255, 0), 2);

        cvLine(pframe, cvPoint(Top_0x,Left_1y), cvPoint(Top_8x,Left_1y), cvScalar(0, 255, 0), 2);
        cvLine(pframe, cvPoint(Top_0x,Left_2y), cvPoint(Top_8x,Left_2y), cvScalar(0, 255, 0), 2);
        cvLine(pframe, cvPoint(Top_0x,Left_3y), cvPoint(Top_8x,Left_3y), cvScalar(0, 255, 0), 2);
        cvLine(pframe, cvPoint(Top_0x,Left_4y), cvPoint(Top_8x,Left_4y), cvScalar(0, 255, 0), 2);
        cvLine(pframe, cvPoint(Top_0x,Left_5y), cvPoint(Top_8x,Left_5y), cvScalar(0, 255, 0), 2);

        cvLine(pframe, cvPoint(Top_0x,Left_6y), cvPoint(Top_8x,Left_6y), cvScalar(0, 255, 0), 2);
        cvLine(pframe, cvPoint(Top_0x,Left_7y), cvPoint(Top_8x,Left_7y), cvScalar(0, 255, 0), 2);

        cvLine(pframe, cvPoint(Top_1x,Left_0y), cvPoint(Top_1x,Left_8y), cvScalar(0, 255, 0), 2);
        cvLine(pframe, cvPoint(Top_2x,Left_0y), cvPoint(Top_2x,Left_8y), cvScalar(0, 255, 0), 2);
        cvLine(pframe, cvPoint(Top_3x,Left_0y), cvPoint(Top_3x,Left_8y), cvScalar(0, 255, 0), 2);
        cvLine(pframe, cvPoint(Top_4x,Left_0y), cvPoint(Top_4x,Left_8y), cvScalar(0, 255, 0), 2);
        cvLine(pframe, cvPoint(Top_5x,Left_0y), cvPoint(Top_5x,Left_8y), cvScalar(0, 255, 0), 2);

        cvLine(pframe, cvPoint(Top_6x,Left_0y), cvPoint(Top_6x,Left_8y), cvScalar(0, 255, 0), 2);
        cvLine(pframe, cvPoint(Top_7x,Left_0y), cvPoint(Top_7x,Left_8y), cvScalar(0, 255, 0), 2);

        if (cstorage == NULL) {
            cstorage = cvCreateMemStorage(0);
        }
        else {
            cvClearMemStorage(cstorage);
        }

        int Nc = cvFindContours(
            flytrack,
            cstorage,
            &contours,
            sizeof(CvContour),
            CV_RETR_EXTERNAL
            );

        for (; contours != 0; contours = contours->h_next) {

            double dArea = 0.0;
            dArea = fabs(cvContourArea(contours,CV_WHOLE_SEQ));


            CvRect r = ((CvContour*)contours)->rect;
            cvMoments(cvGetSubRect(flytrack, &mat, r), &moments, 1);
            M00 = cvGetSpatialMoment(&moments, 0, 0);
            M10 = cvGetSpatialMoment(&moments, 1, 0);
            M01 = cvGetSpatialMoment(&moments, 0, 1);
            centers.x = (int) M10/M00 + r.x;
            centers.y = (int) M01/M00 + r.y;
```

```
cvCircle(pframe, centers, 2, cvScalarAll(255), 1, 8, 0);

if (centers.x > Top_0x && centers.x < Top_1x && centers.y > Left_0y && centers.y < Left_1y)
{   cvPutText(pframe, "1", centers, &font, cvScalar(255, 0, 0));
    fly01area = dArea;
    CV_WRITE_SEQ_ELEM(centers, writer);
}
if (centers.x > Top_0x && centers.x < Top_1x && centers.y > Left_1y && centers.y < Left_2y)
{   cvPutText(pframe, "2", centers, &font, cvScalar(255, 0, 0));
    fly02area = dArea;
    CV_WRITE_SEQ_ELEM(centers, writer);
}
if (centers.x > Top_0x && centers.x < Top_1x && centers.y > Left_2y && centers.y < Left_3y)
{   cvPutText(pframe, "3", centers, &font, cvScalar(255, 0, 0));
    fly03area = dArea;
    CV_WRITE_SEQ_ELEM(centers, writer);
}
if (centers.x > Top_0x && centers.x < Top_1x && centers.y > Left_3y && centers.y < Left_4y)
{   cvPutText(pframe, "4", centers, &font, cvScalar(255, 0, 0));
    fly04area = dArea;
    CV_WRITE_SEQ_ELEM(centers, writer);
}

if (centers.x > Top_0x && centers.x < Top_1x && centers.y > Left_4y && centers.y < Left_5y)
{   cvPutText(pframe, "5", centers, &font, cvScalar(255, 0, 0));
    fly05area = dArea;
    CV_WRITE_SEQ_ELEM(centers, writer);
}

if (centers.x > Top_0x && centers.x < Top_1x && centers.y > Left_5y && centers.y < Left_6y)
{   cvPutText(pframe, "6", centers, &font, cvScalar(255, 0, 0));
    fly06area = dArea;
    CV_WRITE_SEQ_ELEM(centers, writer);
}

if (centers.x > Top_0x && centers.x < Top_1x && centers.y > Left_6y && centers.y < Left_7y)
{   cvPutText(pframe, "7", centers, &font, cvScalar(255, 0, 0));
    fly07area = dArea;
    CV_WRITE_SEQ_ELEM(centers, writer);
}

if (centers.x > Top_0x && centers.x < Top_1x && centers.y > Left_7y && centers.y < Left_8y)
{   cvPutText(pframe, "8", centers, &font, cvScalar(255, 0, 0));
    fly08area = dArea;
    CV_WRITE_SEQ_ELEM(centers, writer);
}



if (centers.x > Top_1x && centers.x < Top_2x && centers.y > Left_0y && centers.y < Left_1y)
{   cvPutText(pframe, "9", centers, &font, cvScalar(0, 255, 0));
    fly09area = dArea;
    CV_WRITE_SEQ_ELEM(centers, writer);
}
if (centers.x > Top_1x && centers.x < Top_2x && centers.y > Left_1y && centers.y < Left_2y)
{   cvPutText(pframe, "10", centers, &font, cvScalar(0, 255, 0));
    fly10area = dArea;
    CV_WRITE_SEQ_ELEM(centers, writer);
}
if (centers.x > Top_1x && centers.x < Top_2x && centers.y > Left_2y && centers.y < Left_3y)
{   cvPutText(pframe, "11", centers, &font, cvScalar(0, 255, 0));
    fly11area = dArea;
    CV_WRITE_SEQ_ELEM(centers, writer);
}
if (centers.x > Top_1x && centers.x < Top_2x && centers.y > Left_3y && centers.y < Left_4y)
{   cvPutText(pframe, "12", centers, &font, cvScalar(0, 255, 0));
    fly12area = dArea;
```

```
        CV_WRITE_SEQ_ELEM(centers, writer);
    }

    if (centers.x > Top_1x && centers.x < Top_2x && centers.y > Left_4y && centers.y < Left_5y)
    {   cvPutText(pframe, "13", centers, &font, cvScalar(0, 255, 0));
        fly13area = dArea;
        CV_WRITE_SEQ_ELEM(centers, writer);
    }

    if (centers.x > Top_1x && centers.x < Top_2x && centers.y > Left_5y && centers.y < Left_6y)
    {   cvPutText(pframe, "14", centers, &font, cvScalar(0, 255, 0));
        fly14area = dArea;
        CV_WRITE_SEQ_ELEM(centers, writer);
    }

    if (centers.x > Top_1x && centers.x < Top_2x && centers.y > Left_6y && centers.y < Left_7y)
    {   cvPutText(pframe, "15", centers, &font, cvScalar(0, 255, 0));
        fly15area = dArea;
        CV_WRITE_SEQ_ELEM(centers, writer);
    }

    if (centers.x > Top_1x && centers.x < Top_2x && centers.y > Left_7y && centers.y < Left_8y)
    {   cvPutText(pframe, "16", centers, &font, cvScalar(0, 255, 0));
        fly16area = dArea;
        CV_WRITE_SEQ_ELEM(centers, writer);
    }



    if (centers.x > Top_2x && centers.x < Top_3x && centers.y > Left_0y && centers.y < Left_1y)
    {   cvPutText(pframe, "17", centers, &font, cvScalar(0, 0, 255));
        fly17area = dArea;
        CV_WRITE_SEQ_ELEM(centers, writer);
    }
    if (centers.x > Top_2x && centers.x < Top_3x && centers.y > Left_1y && centers.y < Left_2y)
    {   cvPutText(pframe, "18", centers, &font, cvScalar(0, 0, 255));
        fly18area = dArea;
        CV_WRITE_SEQ_ELEM(centers, writer);
    }
    if (centers.x > Top_2x && centers.x < Top_3x && centers.y > Left_2y && centers.y < Left_3y)
    {   cvPutText(pframe, "19", centers, &font, cvScalar(0, 0, 255));
        fly19area = dArea;
        CV_WRITE_SEQ_ELEM(centers, writer);
    }
    if (centers.x > Top_2x && centers.x < Top_3x && centers.y > Left_3y && centers.y < Left_4y)
    {   cvPutText(pframe, "20", centers, &font, cvScalar(0, 0, 255));
        fly20area = dArea;
        CV_WRITE_SEQ_ELEM(centers, writer);
    }

    if (centers.x > Top_2x && centers.x < Top_3x && centers.y > Left_4y && centers.y < Left_5y)
    {   cvPutText(pframe, "21", centers, &font, cvScalar(0, 0, 255));
        fly21area = dArea;
        CV_WRITE_SEQ_ELEM(centers, writer);
    }

    if (centers.x > Top_2x && centers.x < Top_3x && centers.y > Left_5y && centers.y < Left_6y)
    {   cvPutText(pframe, "22", centers, &font, cvScalar(0, 0, 255));
        fly22area = dArea;
        CV_WRITE_SEQ_ELEM(centers, writer);
    }

    if (centers.x > Top_2x && centers.x < Top_3x && centers.y > Left_6y && centers.y < Left_7y)
    {   cvPutText(pframe, "23", centers, &font, cvScalar(0, 0, 255));
        fly23area = dArea;
        CV_WRITE_SEQ_ELEM(centers, writer);
    }
```

```
if (centers.x > Top_2x && centers.x < Top_3x && centers.y > Left_7y && centers.y < Left_8y)
{   cvPutText(pframe, "24", centers, &font, cvScalar(0, 0, 255));
    fly24area = dArea;
    CV_WRITE_SEQ_ELEM(centers, writer);
}



if (centers.x > Top_3x && centers.x < Top_4x && centers.y > Left_0y && centers.y < Left_1y)
{   cvPutText(pframe, "25", centers, &font, cvScalar(255, 0, 0));
    fly25area = dArea;
    CV_WRITE_SEQ_ELEM(centers, writer);
}
if (centers.x > Top_3x && centers.x < Top_4x && centers.y > Left_1y && centers.y < Left_2y)
{   cvPutText(pframe, "26", centers, &font, cvScalar(255, 0, 0));
    fly26area = dArea;
    CV_WRITE_SEQ_ELEM(centers, writer);
}
if (centers.x > Top_3x && centers.x < Top_4x && centers.y > Left_2y && centers.y < Left_3y)
{   cvPutText(pframe, "27", centers, &font, cvScalar(255, 0, 0));
    fly27area = dArea;
    CV_WRITE_SEQ_ELEM(centers, writer);
}
if (centers.x > Top_3x && centers.x < Top_4x && centers.y > Left_3y && centers.y < Left_4y)
{   cvPutText(pframe, "28", centers, &font, cvScalar(255, 0, 0));
    fly28area = dArea;
    CV_WRITE_SEQ_ELEM(centers, writer);
}

if (centers.x > Top_3x && centers.x < Top_4x && centers.y > Left_4y && centers.y < Left_5y)
{   cvPutText(pframe, "29", centers, &font, cvScalar(255, 0, 0));
    fly29area = dArea;
    CV_WRITE_SEQ_ELEM(centers, writer);
}

if (centers.x > Top_3x && centers.x < Top_4x && centers.y > Left_5y && centers.y < Left_6y)
{   cvPutText(pframe, "30", centers, &font, cvScalar(255, 0, 0));
    fly30area = dArea;
    CV_WRITE_SEQ_ELEM(centers, writer);
}

if (centers.x > Top_3x && centers.x < Top_4x && centers.y > Left_6y && centers.y < Left_7y)
{   cvPutText(pframe, "31", centers, &font, cvScalar(255, 0, 0));
    fly31area = dArea;
    CV_WRITE_SEQ_ELEM(centers, writer);
}

if (centers.x > Top_3x && centers.x < Top_4x && centers.y > Left_7y && centers.y < Left_8y)
{   cvPutText(pframe, "32", centers, &font, cvScalar(255, 0, 0));
    fly32area = dArea;
    CV_WRITE_SEQ_ELEM(centers, writer);
}



if (centers.x > Top_4x && centers.x < Top_5x && centers.y > Left_0y && centers.y < Left_1y)
{   cvPutText(pframe, "33", centers, &font, cvScalar(0, 255, 0));
    fly33area = dArea;
    CV_WRITE_SEQ_ELEM(centers, writer);
}
if (centers.x > Top_4x && centers.x < Top_5x && centers.y > Left_1y && centers.y < Left_2y)
{   cvPutText(pframe, "34", centers, &font, cvScalar(0, 255, 0));
    fly34area = dArea;
    CV_WRITE_SEQ_ELEM(centers, writer);
}
if (centers.x > Top_4x && centers.x < Top_5x && centers.y > Left_2y && centers.y < Left_3y)
{   cvPutText(pframe, "35", centers, &font, cvScalar(0, 255, 0));
    fly35area = dArea;
```

```
    CV_WRITE_SEQ_ELEM(centers, writer);
}
if (centers.x > Top_4x && centers.x < Top_5x && centers.y > Left_3y && centers.y < Left_4y)
{   cvPutText(pframe, "36", centers, &font, cvScalar(0, 255, 0));
    fly36area = dArea;
    CV_WRITE_SEQ_ELEM(centers, writer);
}

if (centers.x > Top_4x && centers.x < Top_5x && centers.y > Left_4y && centers.y < Left_5y)
{   cvPutText(pframe, "37", centers, &font, cvScalar(0, 255, 0));
    fly37area = dArea;
    CV_WRITE_SEQ_ELEM(centers, writer);
}

if (centers.x > Top_4x && centers.x < Top_5x && centers.y > Left_5y && centers.y < Left_6y)
{   cvPutText(pframe, "38", centers, &font, cvScalar(0, 255, 0));
    fly38area = dArea;
    CV_WRITE_SEQ_ELEM(centers, writer);
}

if (centers.x > Top_4x && centers.x < Top_5x && centers.y > Left_6y && centers.y < Left_7y)
{   cvPutText(pframe, "39", centers, &font, cvScalar(0, 255, 0));
    fly39area = dArea;
    CV_WRITE_SEQ_ELEM(centers, writer);
}

if (centers.x > Top_4x && centers.x < Top_5x && centers.y > Left_7y && centers.y < Left_8y)
{   cvPutText(pframe, "40", centers, &font, cvScalar(0, 255, 0));
    fly40area = dArea;
    CV_WRITE_SEQ_ELEM(centers, writer);
}



if (centers.x > Top_5x && centers.x < Top_6x && centers.y > Left_0y && centers.y < Left_1y)
{   cvPutText(pframe, "41", centers, &font, cvScalar(0, 0, 255));
    fly41area = dArea;
    CV_WRITE_SEQ_ELEM(centers, writer);
}
if (centers.x > Top_5x && centers.x < Top_6x && centers.y > Left_1y && centers.y < Left_2y)
{   cvPutText(pframe, "42", centers, &font, cvScalar(0, 0, 255));
    fly42area = dArea;
    CV_WRITE_SEQ_ELEM(centers, writer);
}
if (centers.x > Top_5x && centers.x < Top_6x && centers.y > Left_2y && centers.y < Left_3y)
{   cvPutText(pframe, "43", centers, &font, cvScalar(0, 0, 255));
    fly43area = dArea;
    CV_WRITE_SEQ_ELEM(centers, writer);
}
if (centers.x > Top_5x && centers.x < Top_6x && centers.y > Left_3y && centers.y < Left_4y)
{   cvPutText(pframe, "44", centers, &font, cvScalar(0, 0, 255));
    fly44area = dArea;
    CV_WRITE_SEQ_ELEM(centers, writer);
}

if (centers.x > Top_5x && centers.x < Top_6x && centers.y > Left_4y && centers.y < Left_5y)
{   cvPutText(pframe, "45", centers, &font, cvScalar(0, 0, 255));
    fly45area = dArea;
    CV_WRITE_SEQ_ELEM(centers, writer);
}

if (centers.x > Top_5x && centers.x < Top_6x && centers.y > Left_5y && centers.y < Left_6y)
{   cvPutText(pframe, "46", centers, &font, cvScalar(0, 0, 255));
    fly46area = dArea;
    CV_WRITE_SEQ_ELEM(centers, writer);
}

if (centers.x > Top_5x && centers.x < Top_6x && centers.y > Left_6y && centers.y < Left_7y)
```

```
{   cvPutText(pframe, "47", centers, &font, cvScalar(0, 0, 255));
    fly47area = dArea;
    CV_WRITE_SEQ_ELEM(centers, writer);
}

if (centers.x > Top_5x && centers.x < Top_6x && centers.y > Left_7y && centers.y < Left_8y)
{   cvPutText(pframe, "48", centers, &font, cvScalar(0, 0, 255));
    fly48area = dArea;
    CV_WRITE_SEQ_ELEM(centers, writer);
}



if (centers.x > Top_6x && centers.x < Top_7x && centers.y > Left_0y && centers.y < Left_1y)
{   cvPutText(pframe, "49", centers, &font, cvScalar(255, 0, 0));
    fly49area = dArea;
    CV_WRITE_SEQ_ELEM(centers, writer);
}
if (centers.x > Top_6x && centers.x < Top_7x && centers.y > Left_1y && centers.y < Left_2y)
{   cvPutText(pframe, "50", centers, &font, cvScalar(255, 0, 0));
    fly50area = dArea;
    CV_WRITE_SEQ_ELEM(centers, writer);
}
if (centers.x > Top_6x && centers.x < Top_7x && centers.y > Left_2y && centers.y < Left_3y)
{   cvPutText(pframe, "51", centers, &font, cvScalar(255, 0, 0));
    fly51area = dArea;
    CV_WRITE_SEQ_ELEM(centers, writer);
}
if (centers.x > Top_6x && centers.x < Top_7x && centers.y > Left_3y && centers.y < Left_4y)
{   cvPutText(pframe, "52", centers, &font, cvScalar(255, 0, 0));
    fly52area = dArea;
    CV_WRITE_SEQ_ELEM(centers, writer);
}

if (centers.x > Top_6x && centers.x < Top_7x && centers.y > Left_4y && centers.y < Left_5y)
{   cvPutText(pframe, "53", centers, &font, cvScalar(255, 0, 0));
    fly53area = dArea;
    CV_WRITE_SEQ_ELEM(centers, writer);
}

if (centers.x > Top_6x && centers.x < Top_7x && centers.y > Left_5y && centers.y < Left_6y)
{   cvPutText(pframe, "54", centers, &font, cvScalar(255, 0, 0));
    fly54area = dArea;
    CV_WRITE_SEQ_ELEM(centers, writer);
}

if (centers.x > Top_6x && centers.x < Top_7x && centers.y > Left_6y && centers.y < Left_7y)
{   cvPutText(pframe, "55", centers, &font, cvScalar(255, 0, 0));
    fly55area = dArea;
    CV_WRITE_SEQ_ELEM(centers, writer);
}

if (centers.x > Top_6x && centers.x < Top_7x && centers.y > Left_7y && centers.y < Left_8y)
{   cvPutText(pframe, "56", centers, &font, cvScalar(255, 0, 0));
    fly56area = dArea;
    CV_WRITE_SEQ_ELEM(centers, writer);
}



if (centers.x > Top_7x && centers.x < Top_8x && centers.y > Left_0y && centers.y < Left_1y)
{   cvPutText(pframe, "57", centers, &font, cvScalar(0, 255, 0));
    fly57area = dArea;
    CV_WRITE_SEQ_ELEM(centers, writer);
}
if (centers.x > Top_7x && centers.x < Top_8x && centers.y > Left_1y && centers.y < Left_2y)
{   cvPutText(pframe, "58", centers, &font, cvScalar(0, 255, 0));
    fly58area = dArea;
```

```
            CV_WRITE_SEQ_ELEM(centers, writer);
        }
        if (centers.x > Top_7x && centers.x < Top_8x && centers.y > Left_2y && centers.y < Left_3y)
        {   cvPutText(pframe, "59", centers, &font, cvScalar(0, 255, 0));
            fly59area = dArea;
            CV_WRITE_SEQ_ELEM(centers, writer);
        }
        if (centers.x > Top_7x && centers.x < Top_8x && centers.y > Left_3y && centers.y < Left_4y)
        {   cvPutText(pframe, "60", centers, &font, cvScalar(0, 255, 0));
            fly60area = dArea;
            CV_WRITE_SEQ_ELEM(centers, writer);
        }

        if (centers.x > Top_7x && centers.x < Top_8x && centers.y > Left_4y && centers.y < Left_5y)
        {   cvPutText(pframe, "61", centers, &font, cvScalar(0, 255, 0));
            fly61area = dArea;
            CV_WRITE_SEQ_ELEM(centers, writer);
        }

        if (centers.x > Top_7x && centers.x < Top_8x && centers.y > Left_5y && centers.y < Left_6y)
        {   cvPutText(pframe, "62", centers, &font, cvScalar(0, 255, 0));
            fly62area = dArea;
            CV_WRITE_SEQ_ELEM(centers, writer);
        }

        if (centers.x > Top_7x && centers.x < Top_8x && centers.y > Left_6y && centers.y < Left_7y)
        {   cvPutText(pframe, "63", centers, &font, cvScalar(0, 255, 0));
            fly63area = dArea;
            CV_WRITE_SEQ_ELEM(centers, writer);
        }

        if (centers.x > Top_7x && centers.x < Top_8x && centers.y > Left_7y && centers.y < Left_8y)
        {   cvPutText(pframe, "64", centers, &font, cvScalar(0, 255, 0));
            fly64area = dArea;
            CV_WRITE_SEQ_ELEM(centers, writer);
        }

    }
    cvFlushSeqWriter(&writer);

    if (psequence->total > Nc * tail_length)
    {
        for (int j = psequence->total - Nc * tail_length - 1; j >= 0; --j)
        {
            cvSeqRemove(psequence, j);
        }
    }

    for (int k = 0; k < psequence->total; ++k)
    {
        CvPoint* p = (CvPoint*)cvGetSeqElem(psequence, k);
        cvCircle(pframe, cvPoint(p->x, p->y), 2, cvScalar(255, 0, 255), 1, 8, 0);

    }

    myfile << "Frame" << i << "\t";
    myfile << "1" << "\t" << fly01area << "\t";
    myfile << "2" << "\t" << fly02area << "\t";
    myfile << "3" << "\t" << fly03area << "\t";
    myfile << "4" << "\t" << fly04area << "\t";
    myfile << "5" << "\t" << fly05area << "\t";
    myfile << "6" << "\t" << fly06area << "\t";
    myfile << "7" << "\t" << fly07area << "\t";
    myfile << "8" << "\t" << fly08area << "\t";
    myfile << "9" << "\t" << fly09area << "\t";
    myfile << "10" << "\t" << fly10area << "\t";
    myfile << "11" << "\t" << fly11area << "\t";
    myfile << "12" << "\t" << fly12area << "\t";
```

```
        myfile << "13" << "\t" << fly13area << "\t";
        myfile << "14" << "\t" << fly14area << "\t";
        myfile << "15" << "\t" << fly15area << "\t";
        myfile << "16" << "\t" << fly16area << "\t";
        myfile << "17" << "\t" << fly17area << "\t";
        myfile << "18" << "\t" << fly18area << "\t";
        myfile << "19" << "\t" << fly19area << "\t";
        myfile << "20" << "\t" << fly20area << "\t";
        myfile << "21" << "\t" << fly21area << "\t";
        myfile << "22" << "\t" << fly22area << "\t";
        myfile << "23" << "\t" << fly23area << "\t";
        myfile << "24" << "\t" << fly24area << "\t";

        myfile << "25" << "\t" << fly25area << "\t";
        myfile << "26" << "\t" << fly26area << "\t";
        myfile << "27" << "\t" << fly27area << "\t";
        myfile << "28" << "\t" << fly28area << "\t";
        myfile << "29" << "\t" << fly29area << "\t";
        myfile << "30" << "\t" << fly30area << "\t";
        myfile << "31" << "\t" << fly31area << "\t";
        myfile << "32" << "\t" << fly32area << "\t";
        myfile << "33" << "\t" << fly33area << "\t";
        myfile << "34" << "\t" << fly34area << "\t";
        myfile << "35" << "\t" << fly35area << "\t";
        myfile << "36" << "\t" << fly36area << "\t";
        myfile << "37" << "\t" << fly37area << "\t";
        myfile << "38" << "\t" << fly38area << "\t";
        myfile << "39" << "\t" << fly39area << "\t";
        myfile << "40" << "\t" << fly40area << "\t";
        myfile << "41" << "\t" << fly41area << "\t";
        myfile << "42" << "\t" << fly42area << "\t";
        myfile << "43" << "\t" << fly43area << "\t";
        myfile << "44" << "\t" << fly44area << "\t";
        myfile << "45" << "\t" << fly45area << "\t";
        myfile << "46" << "\t" << fly46area << "\t";
        myfile << "47" << "\t" << fly47area << "\t";
        myfile << "48" << "\t" << fly48area << "\t";

        myfile << "49" << "\t" << fly49area << "\t";
        myfile << "50" << "\t" << fly50area << "\t";
        myfile << "51" << "\t" << fly51area << "\t";
        myfile << "52" << "\t" << fly52area << "\t";
        myfile << "53" << "\t" << fly53area << "\t";
        myfile << "54" << "\t" << fly54area << "\t";
        myfile << "55" << "\t" << fly55area << "\t";
        myfile << "56" << "\t" << fly56area << "\t";
        myfile << "57" << "\t" << fly57area << "\t";
        myfile << "58" << "\t" << fly58area << "\t";
        myfile << "59" << "\t" << fly59area << "\t";
        myfile << "60" << "\t" << fly60area << "\t";
        myfile << "61" << "\t" << fly61area << "\t";
        myfile << "62" << "\t" << fly62area << "\t";
        myfile << "63" << "\t" << fly63area << "\t";
        myfile << "64" << "\t" << fly64area << "\t";

        myfile << endl;

        cvShowImage("FlyTrack",pframe);

        cout << "Frame #"<< i << " has been analyzed." << endl;

        counter = 0;

        cvWaitKey(30);
    }

}
```

```
        cvEndWriteSeq(&writer);

        cvWaitKey(0);

        myfile.close();
        cvReleaseMemStorage(&cstorage);
        cvReleaseMemStorage(&pstorage);
        cvReleaseImage(&background);
        cvReleaseImage(&flytrack);
        cvReleaseImage(&frame);

        cvDestroyWindow("FlyTrack");
    }

    cvReleaseCapture(&capture);

    return 0;

}
```
