# Supplementary material for: Locomotion Induced by Spatial Restriction in Adult Drosophila
Source: PLoS One. 2015 Sep 9;10(9):e0135825. doi: 10.1371/journal.pone.0135825 (PMC4564261; doi:10.1371/journal.pone.0135825)
Supplement: S1 Appendix — (ZIP) [file pone.0135825.s002.zip › FlyTrack64.pdf]

```cpp
/***********************************************
FlyTrack64.cpp by C. Xiao
***********************************************/
#include "stdafx.h"
#include <iostream>
#include <fstream>
#include <sstream>
#include <string>
#include <cv.h>
#include <highgui.h>
#include <cxcore.h>
using namespace std;

int main(int argc, char** argv)
{
    IplImage*  pframe = NULL;
    IplImage*  frame = NULL;
    IplImage*  background = NULL;
    IplImage*  flytrack = NULL;

    CvCapture* capture = NULL;

    int        start_frame = 0;
    int        total_frames = 0;
    int        analysis_step = 0;
    int        tail_length = 0;

    int        TL_ROIx = 0, TL_ROIy = 0;
    int        BR_ROIx = 0, BR_ROIy = 0;

    int        Top_0x = 0;
    int        Top_1x = 0;
    int        Top_2x = 0;
    int        Top_3x = 0;
    int        Top_4x = 0;
    int        Top_5x = 0;
    int        Top_6x = 0;
    int        Top_7x = 0;
    int        Top_8x = 0;

    int        Left_0y = 0;
    int        Left_1y = 0;
    int        Left_2y = 0;
    int        Left_3y = 0;
    int        Left_4y = 0;
    int        Left_5y = 0;
    int        Left_6y = 0;
    int        Left_7y = 0;
    int        Left_8y = 0;

    int        fly01x = 0, fly01y = 0;
    int        fly02x = 0, fly02y = 0;
    int        fly03x = 0, fly03y = 0;
    int        fly04x = 0, fly04y = 0;
    int        fly05x = 0, fly05y = 0;
    int        fly06x = 0, fly06y = 0;
    int        fly07x = 0, fly07y = 0;
    int        fly08x = 0, fly08y = 0;
    int        fly09x = 0, fly09y = 0;
    int        fly10x = 0, fly10y = 0;
    int        fly11x = 0, fly11y = 0;
    int        fly12x = 0, fly12y = 0;
    int        fly13x = 0, fly13y = 0;
    int        fly14x = 0, fly14y = 0;
    int        fly15x = 0, fly15y = 0;
    int        fly16x = 0, fly16y = 0;
    int        fly17x = 0, fly17y = 0;
    int        fly18x = 0, fly18y = 0;
```

```
int        fly19x = 0, fly19y = 0;
int        fly20x = 0, fly20y = 0;
int        fly21x = 0, fly21y = 0;
int        fly22x = 0, fly22y = 0;
int        fly23x = 0, fly23y = 0;
int        fly24x = 0, fly24y = 0;

int        fly25x = 0, fly25y = 0;
int        fly26x = 0, fly26y = 0;
int        fly27x = 0, fly27y = 0;
int        fly28x = 0, fly28y = 0;
int        fly29x = 0, fly29y = 0;
int        fly30x = 0, fly30y = 0;
int        fly31x = 0, fly31y = 0;
int        fly32x = 0, fly32y = 0;
int        fly33x = 0, fly33y = 0;
int        fly34x = 0, fly34y = 0;
int        fly35x = 0, fly35y = 0;
int        fly36x = 0, fly36y = 0;
int        fly37x = 0, fly37y = 0;
int        fly38x = 0, fly38y = 0;
int        fly39x = 0, fly39y = 0;
int        fly40x = 0, fly40y = 0;
int        fly41x = 0, fly41y = 0;
int        fly42x = 0, fly42y = 0;
int        fly43x = 0, fly43y = 0;
int        fly44x = 0, fly44y = 0;
int        fly45x = 0, fly45y = 0;
int        fly46x = 0, fly46y = 0;
int        fly47x = 0, fly47y = 0;
int        fly48x = 0, fly48y = 0;

int        fly49x = 0, fly49y = 0;
int        fly50x = 0, fly50y = 0;
int        fly51x = 0, fly51y = 0;
int        fly52x = 0, fly52y = 0;
int        fly53x = 0, fly53y = 0;
int        fly54x = 0, fly54y = 0;
int        fly55x = 0, fly55y = 0;
int        fly56x = 0, fly56y = 0;
int        fly57x = 0, fly57y = 0;
int        fly58x = 0, fly58y = 0;
int        fly59x = 0, fly59y = 0;
int        fly60x = 0, fly60y = 0;
int        fly61x = 0, fly617y = 0;
int        fly62x = 0, fly62y = 0;
int        fly63x = 0, fly63y = 0;
int        fly64x = 0, fly64y = 0;

int        counter = 0;

if (argc ==2 && (capture = cvCreateFileCapture(argv[1])) != 0) {

    cout << "Analysis starting at frame (i.e. 1): ";
    cin >> start_frame;

    cout << "Total frames for analysis (i.e. 9000): ";
    cin >> total_frames;

    cout << "Analysis Step (1-15): ";
    cin >> analysis_step;

    cout << "Tracking Tail Length (1-10): ";
    cin >> tail_length;

    cout << "Enter ROI Top Left x Axis Value (in pixel): ";
    cin >> TL_ROIx;
```

```
cout << "Enter ROI Top Left y Axis Value (in pixel): ";
cin >> TL_ROIy;

cout << "Enter ROI Bottom Right x Axis Value (in pixel): ";
cin >> BR_ROIx;

cout << "Enter ROI Bottom Right y Axis Value (in pixel): ";
cin >> BR_ROIy;

Top_0x = 10;
Left_0y = 10;
Top_8x = abs(BR_ROIx - TL_ROIx) - Top_0x;
Left_8y = abs(BR_ROIy - TL_ROIy) - Left_0y;

Top_1x = (int)(Top_0x + (Top_8x - Top_0x)/8);
Top_2x = (int)(Top_0x + 2*(Top_8x - Top_0x)/8);
Top_3x = (int)(Top_0x + 3*(Top_8x - Top_0x)/8);
Top_4x = (int)(Top_0x + 4*(Top_8x - Top_0x)/8);
Top_5x = (int)(Top_0x + 5*(Top_8x - Top_0x)/8);
Top_6x = (int)(Top_0x + 6*(Top_8x - Top_0x)/8);
Top_7x = (int)(Top_0x + 7*(Top_8x - Top_0x)/8);


Left_1y = (int)(Left_0y + (Left_8y - Left_0y)/8);
Left_2y = (int)(Left_0y + 2*(Left_8y - Left_0y)/8);
Left_3y = (int)(Left_0y + 3*(Left_8y - Left_0y)/8);
Left_4y = (int)(Left_0y + 4*(Left_8y - Left_0y)/8);
Left_5y = (int)(Left_0y + 5*(Left_8y - Left_0y)/8);
Left_6y = (int)(Left_0y + 6*(Left_8y - Left_0y)/8);
Left_7y = (int)(Left_0y + 7*(Left_8y - Left_0y)/8);

background = cvLoadImage("Background.jpg", 0);
cvSetImageROI(background, cvRect(TL_ROIx, TL_ROIy, abs(BR_ROIx - TL_ROIx), abs(BR_ROIy - TL_ROIy)));

flytrack = cvCloneImage(background);
frame = cvCloneImage(background);
cvZero(flytrack);
cvZero(frame);

cvNamedWindow("FlyTrack",1);

CvMemStorage* cstorage = NULL;
CvSeq*        contours = NULL;

CvMemStorage* pstorage = NULL;
pstorage = cvCreateMemStorage(0);

CvSeqWriter   writer;
CvSeq*        psequence = NULL;

cvStartWriteSeq(CV_32SC2, sizeof(CvSeq), sizeof(CvPoint), pstorage, &writer);
psequence = cvEndWriteSeq(&writer);

cvStartAppendToSeq(psequence, &writer);

CvMoments moments;
double M00, M01, M10;
CvPoint centers;
CvMat mat;

CvFont font;
cvInitFont(&font, CV_FONT_HERSHEY_PLAIN, 1, 2, 0, 1, 8);

ofstream myfile;
myfile.open("Results.txt");

for (int i = 0; i < start_frame; i++)
{
```

```
        pframe = cvQueryFrame(capture);
    }

    for( int i = start_frame; i <= start_frame + total_frames; i++) {

        pframe = cvQueryFrame(capture);
        counter ++;

        if (counter == analysis_step) {

            cvSetImageROI(pframe, cvRect(TL_ROIx, TL_ROIy, abs(BR_ROIx - TL_ROIx), abs(BR_ROIy - TL_ROIy)));

            cvCvtColor(pframe, frame, CV_BGR2GRAY);

            cvAbsDiff(frame, background, flytrack);
            cvThreshold(flytrack, flytrack, 60, 255, CV_THRESH_BINARY);

            cvMorphologyEx(flytrack, flytrack, 0, 0, CV_MOP_OPEN, 1);
            cvMorphologyEx(flytrack, flytrack, 0, 0, CV_MOP_CLOSE, 1);

            cvErode(flytrack, flytrack, 0, 1);
            cvDilate(flytrack, flytrack, 0, 3);

            cvSmooth(flytrack, flytrack, CV_MEDIAN, 3, 3);

            cvRectangle(pframe, cvPoint(Top_0x, Left_0y), cvPoint(Top_8x,Left_8y), cvScalar(0, 255, 0), 2);

            cvLine(pframe, cvPoint(Top_0x,Left_1y), cvPoint(Top_8x,Left_1y), cvScalar(0, 255, 0), 2);
            cvLine(pframe, cvPoint(Top_0x,Left_2y), cvPoint(Top_8x,Left_2y), cvScalar(0, 255, 0), 2);
            cvLine(pframe, cvPoint(Top_0x,Left_3y), cvPoint(Top_8x,Left_3y), cvScalar(0, 255, 0), 2);
            cvLine(pframe, cvPoint(Top_0x,Left_4y), cvPoint(Top_8x,Left_4y), cvScalar(0, 255, 0), 2);
            cvLine(pframe, cvPoint(Top_0x,Left_5y), cvPoint(Top_8x,Left_5y), cvScalar(0, 255, 0), 2);
            cvLine(pframe, cvPoint(Top_0x,Left_6y), cvPoint(Top_8x,Left_6y), cvScalar(0, 255, 0), 2);
            cvLine(pframe, cvPoint(Top_0x,Left_7y), cvPoint(Top_8x,Left_7y), cvScalar(0, 255, 0), 2);

            cvLine(pframe, cvPoint(Top_1x,Left_0y), cvPoint(Top_1x,Left_8y), cvScalar(0, 255, 0), 2);
            cvLine(pframe, cvPoint(Top_2x,Left_0y), cvPoint(Top_2x,Left_8y), cvScalar(0, 255, 0), 2);
            cvLine(pframe, cvPoint(Top_3x,Left_0y), cvPoint(Top_3x,Left_8y), cvScalar(0, 255, 0), 2);
            cvLine(pframe, cvPoint(Top_4x,Left_0y), cvPoint(Top_4x,Left_8y), cvScalar(0, 255, 0), 2);
            cvLine(pframe, cvPoint(Top_5x,Left_0y), cvPoint(Top_5x,Left_8y), cvScalar(0, 255, 0), 2);
            cvLine(pframe, cvPoint(Top_6x,Left_0y), cvPoint(Top_6x,Left_8y), cvScalar(0, 255, 0), 2);
            cvLine(pframe, cvPoint(Top_7x,Left_0y), cvPoint(Top_7x,Left_8y), cvScalar(0, 255, 0), 2);


            if (cstorage == NULL) {
                cstorage = cvCreateMemStorage(0);
            }
            else {
                cvClearMemStorage(cstorage);
            }

            int Nc = cvFindContours(
                flytrack,
                cstorage,
                &contours,
                sizeof(CvContour),
                CV_RETR_EXTERNAL
                );

            for (; contours != 0; contours = contours->h_next) {

                CvRect r = ((CvContour*)contours)->rect;
                cvMoments(cvGetSubRect(flytrack, &mat, r), &moments, 1);
                M00 = cvGetSpatialMoment(&moments, 0, 0);
                M10 = cvGetSpatialMoment(&moments, 1, 0);
                M01 = cvGetSpatialMoment(&moments, 0, 1);
                centers.x = (int) M10/M00 + r.x;
                centers.y = (int) M01/M00 + r.y;
```

```
cvCircle(pframe, centers, 2, cvScalarAll(255), 1, 8, 0);

if (centers.x > Top_0x && centers.x < Top_1x && centers.y > Left_0y && centers.y < Left_1y)
{   cvPutText(pframe, "1", centers, &font, cvScalar(255, 0, 0));
    fly01x = centers.x; fly01y = centers.y;
    CV_WRITE_SEQ_ELEM(centers, writer);
}
if (centers.x > Top_0x && centers.x < Top_1x && centers.y > Left_1y && centers.y < Left_2y)
{   cvPutText(pframe, "2", centers, &font, cvScalar(255, 0, 0));
    fly02x = centers.x; fly02y = centers.y;
    CV_WRITE_SEQ_ELEM(centers, writer);
}
if (centers.x > Top_0x && centers.x < Top_1x && centers.y > Left_2y && centers.y < Left_3y)
{   cvPutText(pframe, "3", centers, &font, cvScalar(255, 0, 0));
    fly03x = centers.x; fly03y = centers.y;
    CV_WRITE_SEQ_ELEM(centers, writer);
}
if (centers.x > Top_0x && centers.x < Top_1x && centers.y > Left_3y && centers.y < Left_4y)
{   cvPutText(pframe, "4", centers, &font, cvScalar(255, 0, 0));
    fly04x = centers.x; fly04y = centers.y;
    CV_WRITE_SEQ_ELEM(centers, writer);
}

if (centers.x > Top_0x && centers.x < Top_1x && centers.y > Left_4y && centers.y < Left_5y)
{   cvPutText(pframe, "5", centers, &font, cvScalar(255, 0, 0));
    fly05x = centers.x; fly05y = centers.y;
    CV_WRITE_SEQ_ELEM(centers, writer);
}

if (centers.x > Top_0x && centers.x < Top_1x && centers.y > Left_5y && centers.y < Left_6y)
{   cvPutText(pframe, "6", centers, &font, cvScalar(255, 0, 0));
    fly06x = centers.x; fly06y = centers.y;
    CV_WRITE_SEQ_ELEM(centers, writer);
}
if (centers.x > Top_0x && centers.x < Top_1x && centers.y > Left_6y && centers.y < Left_7y)
{   cvPutText(pframe, "7", centers, &font, cvScalar(255, 0, 0));
    fly07x = centers.x; fly07y = centers.y;
    CV_WRITE_SEQ_ELEM(centers, writer);
}
if (centers.x > Top_0x && centers.x < Top_1x && centers.y > Left_7y && centers.y < Left_8y)
{   cvPutText(pframe, "8", centers, &font, cvScalar(255, 0, 0));
    fly08x = centers.x; fly08y = centers.y;
    CV_WRITE_SEQ_ELEM(centers, writer);
}


if (centers.x > Top_1x && centers.x < Top_2x && centers.y > Left_0y && centers.y < Left_1y)
{   cvPutText(pframe, "9", centers, &font, cvScalar(0, 255, 0));
    fly09x = centers.x; fly09y = centers.y;
    CV_WRITE_SEQ_ELEM(centers, writer);
}
if (centers.x > Top_1x && centers.x < Top_2x && centers.y > Left_1y && centers.y < Left_2y)
{   cvPutText(pframe, "10", centers, &font, cvScalar(0, 255, 0));
    fly10x = centers.x; fly10y = centers.y;
    CV_WRITE_SEQ_ELEM(centers, writer);
}
if (centers.x > Top_1x && centers.x < Top_2x && centers.y > Left_2y && centers.y < Left_3y)
{   cvPutText(pframe, "11", centers, &font, cvScalar(0, 255, 0));
    fly11x = centers.x; fly11y = centers.y;
    CV_WRITE_SEQ_ELEM(centers, writer);
}
if (centers.x > Top_1x && centers.x < Top_2x && centers.y > Left_3y && centers.y < Left_4y)
{   cvPutText(pframe, "12", centers, &font, cvScalar(0, 255, 0));
    fly12x = centers.x; fly12y = centers.y;
    CV_WRITE_SEQ_ELEM(centers, writer);
}
```

```
if (centers.x > Top_1x && centers.x < Top_2x && centers.y > Left_4y && centers.y < Left_5y)
{   cvPutText(pframe, "13", centers, &font, cvScalar(0, 255, 0));
    fly13x = centers.x; fly13y = centers.y;
    CV_WRITE_SEQ_ELEM(centers, writer);
}

if (centers.x > Top_1x && centers.x < Top_2x && centers.y > Left_5y && centers.y < Left_6y)
{   cvPutText(pframe, "14", centers, &font, cvScalar(0, 255, 0));
    fly14x = centers.x; fly14y = centers.y;
    CV_WRITE_SEQ_ELEM(centers, writer);
}
if (centers.x > Top_1x && centers.x < Top_2x && centers.y > Left_6y && centers.y < Left_7y)
{   cvPutText(pframe, "15", centers, &font, cvScalar(0, 255, 0));
    fly15x = centers.x; fly15y = centers.y;
    CV_WRITE_SEQ_ELEM(centers, writer);
}
if (centers.x > Top_1x && centers.x < Top_2x && centers.y > Left_7y && centers.y < Left_8y)
{   cvPutText(pframe, "16", centers, &font, cvScalar(0, 255, 0));
    fly16x = centers.x; fly16y = centers.y;
    CV_WRITE_SEQ_ELEM(centers, writer);
}



if (centers.x > Top_2x && centers.x < Top_3x && centers.y > Left_0y && centers.y < Left_1y)
{   cvPutText(pframe, "17", centers, &font, cvScalar(0, 0, 255));
    fly17x = centers.x; fly17y = centers.y;
    CV_WRITE_SEQ_ELEM(centers, writer);
}
if (centers.x > Top_2x && centers.x < Top_3x && centers.y > Left_1y && centers.y < Left_2y)
{   cvPutText(pframe, "18", centers, &font, cvScalar(0, 0, 255));
    fly18x = centers.x; fly18y = centers.y;
    CV_WRITE_SEQ_ELEM(centers, writer);
}
if (centers.x > Top_2x && centers.x < Top_3x && centers.y > Left_2y && centers.y < Left_3y)
{   cvPutText(pframe, "19", centers, &font, cvScalar(0, 0, 255));
    fly19x = centers.x; fly19y = centers.y;
    CV_WRITE_SEQ_ELEM(centers, writer);
}
if (centers.x > Top_2x && centers.x < Top_3x && centers.y > Left_3y && centers.y < Left_4y)
{   cvPutText(pframe, "20", centers, &font, cvScalar(0, 0, 255));
    fly20x = centers.x; fly20y = centers.y;
    CV_WRITE_SEQ_ELEM(centers, writer);
}

if (centers.x > Top_2x && centers.x < Top_3x && centers.y > Left_4y && centers.y < Left_5y)
{   cvPutText(pframe, "21", centers, &font, cvScalar(0, 0, 255));
    fly21x = centers.x; fly21y = centers.y;
    CV_WRITE_SEQ_ELEM(centers, writer);
}

if (centers.x > Top_2x && centers.x < Top_3x && centers.y > Left_5y && centers.y < Left_6y)
{   cvPutText(pframe, "22", centers, &font, cvScalar(0, 0, 255));
    fly22x = centers.x; fly22y = centers.y;
    CV_WRITE_SEQ_ELEM(centers, writer);
}
if (centers.x > Top_2x && centers.x < Top_3x && centers.y > Left_6y && centers.y < Left_7y)
{   cvPutText(pframe, "23", centers, &font, cvScalar(0, 0, 255));
    fly23x = centers.x; fly23y = centers.y;
    CV_WRITE_SEQ_ELEM(centers, writer);
}
if (centers.x > Top_2x && centers.x < Top_3x && centers.y > Left_7y && centers.y < Left_8y)
{   cvPutText(pframe, "24", centers, &font, cvScalar(0, 0, 255));
    fly24x = centers.x; fly24y = centers.y;
    CV_WRITE_SEQ_ELEM(centers, writer);
}
```

```
if (centers.x > Top_3x && centers.x < Top_4x && centers.y > Left_0y && centers.y < Left_1y)
{   cvPutText(pframe, "25", centers, &font, cvScalar(255, 0, 0));
    fly25x = centers.x; fly25y = centers.y;
    CV_WRITE_SEQ_ELEM(centers, writer);
}
if (centers.x > Top_3x && centers.x < Top_4x && centers.y > Left_1y && centers.y < Left_2y)
{   cvPutText(pframe, "26", centers, &font, cvScalar(255, 0, 0));
    fly26x = centers.x; fly26y = centers.y;
    CV_WRITE_SEQ_ELEM(centers, writer);
}
if (centers.x > Top_3x && centers.x < Top_4x && centers.y > Left_2y && centers.y < Left_3y)
{   cvPutText(pframe, "27", centers, &font, cvScalar(255, 0, 0));
    fly27x = centers.x; fly27y = centers.y;
    CV_WRITE_SEQ_ELEM(centers, writer);
}
if (centers.x > Top_3x && centers.x < Top_4x && centers.y > Left_3y && centers.y < Left_4y)
{   cvPutText(pframe, "28", centers, &font, cvScalar(255, 0, 0));
    fly28x = centers.x; fly28y = centers.y;
    CV_WRITE_SEQ_ELEM(centers, writer);
}

if (centers.x > Top_3x && centers.x < Top_4x && centers.y > Left_4y && centers.y < Left_5y)
{   cvPutText(pframe, "29", centers, &font, cvScalar(255, 0, 0));
    fly29x = centers.x; fly29y = centers.y;
    CV_WRITE_SEQ_ELEM(centers, writer);
}

if (centers.x > Top_3x && centers.x < Top_4x && centers.y > Left_5y && centers.y < Left_6y)
{   cvPutText(pframe, "30", centers, &font, cvScalar(255, 0, 0));
    fly30x = centers.x; fly30y = centers.y;
    CV_WRITE_SEQ_ELEM(centers, writer);
}
if (centers.x > Top_3x && centers.x < Top_4x && centers.y > Left_6y && centers.y < Left_7y)
{   cvPutText(pframe, "31", centers, &font, cvScalar(255, 0, 0));
    fly31x = centers.x; fly31y = centers.y;
    CV_WRITE_SEQ_ELEM(centers, writer);
}
if (centers.x > Top_3x && centers.x < Top_4x && centers.y > Left_7y && centers.y < Left_8y)
{   cvPutText(pframe, "32", centers, &font, cvScalar(255, 0, 0));
    fly32x = centers.x; fly32y = centers.y;
    CV_WRITE_SEQ_ELEM(centers, writer);
}


if (centers.x > Top_4x && centers.x < Top_5x && centers.y > Left_0y && centers.y < Left_1y)
{   cvPutText(pframe, "33", centers, &font, cvScalar(0, 255, 0));
    fly33x = centers.x; fly33y = centers.y;
    CV_WRITE_SEQ_ELEM(centers, writer);
}
if (centers.x > Top_4x && centers.x < Top_5x && centers.y > Left_1y && centers.y < Left_2y)
{   cvPutText(pframe, "34", centers, &font, cvScalar(0, 255, 0));
    fly34x = centers.x; fly34y = centers.y;
    CV_WRITE_SEQ_ELEM(centers, writer);
}
if (centers.x > Top_4x && centers.x < Top_5x && centers.y > Left_2y && centers.y < Left_3y)
{   cvPutText(pframe, "35", centers, &font, cvScalar(0, 255, 0));
    fly35x = centers.x; fly35y = centers.y;
    CV_WRITE_SEQ_ELEM(centers, writer);
}
if (centers.x > Top_4x && centers.x < Top_5x && centers.y > Left_3y && centers.y < Left_4y)
{   cvPutText(pframe, "36", centers, &font, cvScalar(0, 255, 0));
    fly36x = centers.x; fly36y = centers.y;
    CV_WRITE_SEQ_ELEM(centers, writer);
}

if (centers.x > Top_4x && centers.x < Top_5x && centers.y > Left_4y && centers.y < Left_5y)
{   cvPutText(pframe, "37", centers, &font, cvScalar(0, 255, 0));
    fly37x = centers.x; fly37y = centers.y;
```

```
        CV_WRITE_SEQ_ELEM(centers, writer);
    }

    if (centers.x > Top_4x && centers.x < Top_5x && centers.y > Left_5y && centers.y < Left_6y)
    {   cvPutText(pframe, "38", centers, &font, cvScalar(0, 255, 0));
        fly38x = centers.x; fly38y = centers.y;
        CV_WRITE_SEQ_ELEM(centers, writer);
    }
    if (centers.x > Top_4x && centers.x < Top_5x && centers.y > Left_6y && centers.y < Left_7y)
    {   cvPutText(pframe, "39", centers, &font, cvScalar(0, 255, 0));
        fly39x = centers.x; fly39y = centers.y;
        CV_WRITE_SEQ_ELEM(centers, writer);
    }
    if (centers.x > Top_4x && centers.x < Top_5x && centers.y > Left_7y && centers.y < Left_8y)
    {   cvPutText(pframe, "40", centers, &font, cvScalar(0, 255, 0));
        fly40x = centers.x; fly40y = centers.y;
        CV_WRITE_SEQ_ELEM(centers, writer);
    }


    if (centers.x > Top_5x && centers.x < Top_6x && centers.y > Left_0y && centers.y < Left_1y)
    {   cvPutText(pframe, "41", centers, &font, cvScalar(0, 0, 255));
        fly41x = centers.x; fly41y = centers.y;
        CV_WRITE_SEQ_ELEM(centers, writer);
    }
    if (centers.x > Top_5x && centers.x < Top_6x && centers.y > Left_1y && centers.y < Left_2y)
    {   cvPutText(pframe, "42", centers, &font, cvScalar(0, 0, 255));
        fly42x = centers.x; fly42y = centers.y;
        CV_WRITE_SEQ_ELEM(centers, writer);
    }
    if (centers.x > Top_5x && centers.x < Top_6x && centers.y > Left_2y && centers.y < Left_3y)
    {   cvPutText(pframe, "43", centers, &font, cvScalar(0, 0, 255));
        fly43x = centers.x; fly43y = centers.y;
        CV_WRITE_SEQ_ELEM(centers, writer);
    }
    if (centers.x > Top_5x && centers.x < Top_6x && centers.y > Left_3y && centers.y < Left_4y)
    {   cvPutText(pframe, "44", centers, &font, cvScalar(0, 0, 255));
        fly44x = centers.x; fly44y = centers.y;
        CV_WRITE_SEQ_ELEM(centers, writer);
    }

    if (centers.x > Top_5x && centers.x < Top_6x && centers.y > Left_4y && centers.y < Left_5y)
    {   cvPutText(pframe, "45", centers, &font, cvScalar(0, 0, 255));
        fly45x = centers.x; fly45y = centers.y;
        CV_WRITE_SEQ_ELEM(centers, writer);
    }

    if (centers.x > Top_5x && centers.x < Top_6x && centers.y > Left_5y && centers.y < Left_6y)
    {   cvPutText(pframe, "46", centers, &font, cvScalar(0, 0, 255));
        fly46x = centers.x; fly46y = centers.y;
        CV_WRITE_SEQ_ELEM(centers, writer);
    }
    if (centers.x > Top_5x && centers.x < Top_6x && centers.y > Left_6y && centers.y < Left_7y)
    {   cvPutText(pframe, "47", centers, &font, cvScalar(0, 0, 255));
        fly47x = centers.x; fly47y = centers.y;
        CV_WRITE_SEQ_ELEM(centers, writer);
    }
    if (centers.x > Top_5x && centers.x < Top_6x && centers.y > Left_7y && centers.y < Left_8y)
    {   cvPutText(pframe, "48", centers, &font, cvScalar(0, 0, 255));
        fly48x = centers.x; fly48y = centers.y;
        CV_WRITE_SEQ_ELEM(centers, writer);
    }


    if (centers.x > Top_6x && centers.x < Top_7x && centers.y > Left_0y && centers.y < Left_1y)
    {   cvPutText(pframe, "49", centers, &font, cvScalar(0, 0, 255));
        fly49x = centers.x; fly49y = centers.y;
        CV_WRITE_SEQ_ELEM(centers, writer);
```

```
}
if (centers.x > Top_6x && centers.x < Top_7x && centers.y > Left_1y && centers.y < Left_2y)
{   cvPutText(pframe, "50", centers, &font, cvScalar(0, 0, 255));
    fly50x = centers.x; fly50y = centers.y;
    CV_WRITE_SEQ_ELEM(centers, writer);
}
if (centers.x > Top_6x && centers.x < Top_7x && centers.y > Left_2y && centers.y < Left_3y)
{   cvPutText(pframe, "51", centers, &font, cvScalar(0, 0, 255));
    fly51x = centers.x; fly51y = centers.y;
    CV_WRITE_SEQ_ELEM(centers, writer);
}
if (centers.x > Top_6x && centers.x < Top_7x && centers.y > Left_3y && centers.y < Left_4y)
{   cvPutText(pframe, "52", centers, &font, cvScalar(0, 0, 255));
    fly52x = centers.x; fly52y = centers.y;
    CV_WRITE_SEQ_ELEM(centers, writer);
}

if (centers.x > Top_6x && centers.x < Top_7x && centers.y > Left_4y && centers.y < Left_5y)
{   cvPutText(pframe, "53", centers, &font, cvScalar(0, 0, 255));
    fly53x = centers.x; fly53y = centers.y;
    CV_WRITE_SEQ_ELEM(centers, writer);
}

if (centers.x > Top_6x && centers.x < Top_7x && centers.y > Left_5y && centers.y < Left_6y)
{   cvPutText(pframe, "54", centers, &font, cvScalar(0, 0, 255));
    fly54x = centers.x; fly54y = centers.y;
    CV_WRITE_SEQ_ELEM(centers, writer);
}
if (centers.x > Top_6x && centers.x < Top_7x && centers.y > Left_6y && centers.y < Left_7y)
{   cvPutText(pframe, "55", centers, &font, cvScalar(0, 0, 255));
    fly55x = centers.x; fly55y = centers.y;
    CV_WRITE_SEQ_ELEM(centers, writer);
}
if (centers.x > Top_6x && centers.x < Top_7x && centers.y > Left_7y && centers.y < Left_8y)
{   cvPutText(pframe, "56", centers, &font, cvScalar(0, 0, 255));
    fly56x = centers.x; fly56y = centers.y;
    CV_WRITE_SEQ_ELEM(centers, writer);
}


if (centers.x > Top_7x && centers.x < Top_8x && centers.y > Left_0y && centers.y < Left_1y)
{   cvPutText(pframe, "57", centers, &font, cvScalar(0, 0, 255));
    fly57x = centers.x; fly57y = centers.y;
    CV_WRITE_SEQ_ELEM(centers, writer);
}
if (centers.x > Top_7x && centers.x < Top_8x && centers.y > Left_1y && centers.y < Left_2y)
{   cvPutText(pframe, "58", centers, &font, cvScalar(0, 0, 255));
    fly58x = centers.x; fly58y = centers.y;
    CV_WRITE_SEQ_ELEM(centers, writer);
}
if (centers.x > Top_7x && centers.x < Top_8x && centers.y > Left_2y && centers.y < Left_3y)
{   cvPutText(pframe, "59", centers, &font, cvScalar(0, 0, 255));
    fly59x = centers.x; fly59y = centers.y;
    CV_WRITE_SEQ_ELEM(centers, writer);
}
if (centers.x > Top_7x && centers.x < Top_8x && centers.y > Left_3y && centers.y < Left_4y)
{   cvPutText(pframe, "60", centers, &font, cvScalar(0, 0, 255));
    fly60x = centers.x; fly60y = centers.y;
    CV_WRITE_SEQ_ELEM(centers, writer);
}

if (centers.x > Top_7x && centers.x < Top_8x && centers.y > Left_4y && centers.y < Left_5y)
{   cvPutText(pframe, "61", centers, &font, cvScalar(0, 0, 255));
    fly61x = centers.x; fly61y = centers.y;
    CV_WRITE_SEQ_ELEM(centers, writer);
}

if (centers.x > Top_7x && centers.x < Top_8x && centers.y > Left_5y && centers.y < Left_6y)
```

```
    {   cvPutText(pframe, "62", centers, &font, cvScalar(0, 0, 255));
        fly62x = centers.x; fly62y = centers.y;
        CV_WRITE_SEQ_ELEM(centers, writer);
    }
    if (centers.x > Top_7x && centers.x < Top_8x && centers.y > Left_6y && centers.y < Left_7y)
    {   cvPutText(pframe, "63", centers, &font, cvScalar(0, 0, 255));
        fly63x = centers.x; fly63y = centers.y;
        CV_WRITE_SEQ_ELEM(centers, writer);
    }
    if (centers.x > Top_7x && centers.x < Top_8x && centers.y > Left_7y && centers.y < Left_8y)
    {   cvPutText(pframe, "64", centers, &font, cvScalar(0, 0, 255));
        fly64x = centers.x; fly64y = centers.y;
        CV_WRITE_SEQ_ELEM(centers, writer);
    }




}
cvFlushSeqWriter(&writer);

if (psequence->total > Nc * tail_length)
{
    for (int j = psequence->total - Nc * tail_length - 1; j >= 0; --j)
    {
        cvSeqRemove(psequence, j);
    }
}

for (int k = 0; k < psequence->total; ++k)
{
    CvPoint* p = (CvPoint*)cvGetSeqElem(psequence, k);
    cvCircle(pframe, cvPoint(p->x, p->y), 2, cvScalar(255, 0, 255), 1, 8, 0);

}

myfile << "Frame" << i << "\t";
myfile << "1" << "\t" << fly01x << "\t" << fly01y << "\t";
myfile << "2" << "\t" << fly02x << "\t" << fly02y << "\t";
myfile << "3" << "\t" << fly03x << "\t" << fly03y << "\t";
myfile << "4" << "\t" << fly04x << "\t" << fly04y << "\t";
myfile << "5" << "\t" << fly05x << "\t" << fly05y << "\t";
myfile << "6" << "\t" << fly06x << "\t" << fly06y << "\t";
myfile << "7" << "\t" << fly07x << "\t" << fly07y << "\t";
myfile << "8" << "\t" << fly08x << "\t" << fly08y << "\t";
myfile << "9" << "\t" << fly09x << "\t" << fly09y << "\t";
myfile << "10" << "\t" << fly10x << "\t" << fly10y << "\t";
myfile << "11" << "\t" << fly11x << "\t" << fly11y << "\t";
myfile << "12" << "\t" << fly12x << "\t" << fly12y << "\t";
myfile << "13" << "\t" << fly13x << "\t" << fly13y << "\t";
myfile << "14" << "\t" << fly14x << "\t" << fly14y << "\t";
myfile << "15" << "\t" << fly15x << "\t" << fly15y << "\t";
myfile << "16" << "\t" << fly16x << "\t" << fly16y << "\t";
myfile << "17" << "\t" << fly17x << "\t" << fly17y << "\t";
myfile << "18" << "\t" << fly18x << "\t" << fly18y << "\t";
myfile << "19" << "\t" << fly19x << "\t" << fly19y << "\t";
myfile << "20" << "\t" << fly20x << "\t" << fly20y << "\t";
myfile << "21" << "\t" << fly21x << "\t" << fly21y << "\t";
myfile << "22" << "\t" << fly22x << "\t" << fly22y << "\t";
myfile << "23" << "\t" << fly23x << "\t" << fly23y << "\t";
myfile << "24" << "\t" << fly24x << "\t" << fly24y << "\t";

myfile << "25" << "\t" << fly25x << "\t" << fly25y << "\t";
myfile << "26" << "\t" << fly26x << "\t" << fly26y << "\t";
myfile << "27" << "\t" << fly27x << "\t" << fly27y << "\t";
myfile << "28" << "\t" << fly28x << "\t" << fly28y << "\t";
myfile << "29" << "\t" << fly29x << "\t" << fly29y << "\t";
myfile << "30" << "\t" << fly30x << "\t" << fly30y << "\t";
```

```
            myfile << "31" << "\t" << fly31x << "\t" << fly31y << "\t";
            myfile << "32" << "\t" << fly32x << "\t" << fly32y << "\t";
            myfile << "33" << "\t" << fly33x << "\t" << fly33y << "\t";
            myfile << "34" << "\t" << fly34x << "\t" << fly34y << "\t";
            myfile << "35" << "\t" << fly35x << "\t" << fly35y << "\t";
            myfile << "36" << "\t" << fly36x << "\t" << fly36y << "\t";
            myfile << "37" << "\t" << fly37x << "\t" << fly37y << "\t";
            myfile << "38" << "\t" << fly38x << "\t" << fly38y << "\t";
            myfile << "39" << "\t" << fly39x << "\t" << fly39y << "\t";
            myfile << "40" << "\t" << fly40x << "\t" << fly40y << "\t";
            myfile << "41" << "\t" << fly41x << "\t" << fly41y << "\t";
            myfile << "42" << "\t" << fly42x << "\t" << fly42y << "\t";
            myfile << "43" << "\t" << fly43x << "\t" << fly43y << "\t";
            myfile << "44" << "\t" << fly44x << "\t" << fly44y << "\t";
            myfile << "45" << "\t" << fly45x << "\t" << fly45y << "\t";
            myfile << "46" << "\t" << fly46x << "\t" << fly46y << "\t";
            myfile << "47" << "\t" << fly47x << "\t" << fly47y << "\t";
            myfile << "48" << "\t" << fly48x << "\t" << fly48y << "\t";

            myfile << "49" << "\t" << fly49x << "\t" << fly49y << "\t";
            myfile << "50" << "\t" << fly50x << "\t" << fly50y << "\t";
            myfile << "51" << "\t" << fly51x << "\t" << fly51y << "\t";
            myfile << "52" << "\t" << fly52x << "\t" << fly52y << "\t";
            myfile << "53" << "\t" << fly53x << "\t" << fly53y << "\t";
            myfile << "54" << "\t" << fly54x << "\t" << fly54y << "\t";
            myfile << "55" << "\t" << fly55x << "\t" << fly55y << "\t";
            myfile << "56" << "\t" << fly56x << "\t" << fly56y << "\t";
            myfile << "57" << "\t" << fly57x << "\t" << fly57y << "\t";
            myfile << "58" << "\t" << fly58x << "\t" << fly58y << "\t";
            myfile << "59" << "\t" << fly59x << "\t" << fly59y << "\t";
            myfile << "60" << "\t" << fly60x << "\t" << fly60y << "\t";
            myfile << "61" << "\t" << fly61x << "\t" << fly61y << "\t";
            myfile << "62" << "\t" << fly62x << "\t" << fly62y << "\t";
            myfile << "63" << "\t" << fly63x << "\t" << fly63y << "\t";
            myfile << "64" << "\t" << fly64x << "\t" << fly64y;

            myfile << endl;

            cvShowImage("FlyTrack",pframe);

            cout << "Frame #"<< i << " has been analyzed." << endl;

            counter = 0;

            cvWaitKey(30);
        }

    }

    cvEndWriteSeq(&writer);

    cvWaitKey(0);

    myfile.close();
    cvReleaseMemStorage(&cstorage);
    cvReleaseMemStorage(&pstorage);
    cvReleaseImage(&background);
    cvReleaseImage(&flytrack);
    cvReleaseImage(&frame);

    cvDestroyWindow("FlyTrack");
}

cvReleaseCapture(&capture);

return 0;

}
```
