# Supplementary material for: Locomotion Induced by Spatial Restriction in Adult Drosophila
Source: PLoS One. 2015 Sep 9;10(9):e0135825. doi: 10.1371/journal.pone.0135825 (PMC4564261; doi:10.1371/journal.pone.0135825)
Supplement: S1 Appendix — (ZIP) [file pone.0135825.s002.zip › FlyTrack9.pdf]

```
/**********************************************
FlyTrack9.cpp
**********************************************/
#include <stdafx.h>
#include <iostream>
#include <fstream>
#include <sstream>
#include <string>
#include <cv.h>
#include <highgui.h>
#include <cxcore.h>
using namespace std;

int main(int argc, char** argv)
{
    IplImage*  pframe = NULL;
    IplImage*  frame = NULL;
    IplImage*  background = NULL;
    IplImage*  flytrack = NULL;

    CvCapture* capture = NULL;

    int        start_frame = 0;
    int        total_frames = 0;
    int        analysis_step = 0;
    int        tail_length = 0;

    int        TL_ROIx = 0, TL_ROIy = 0;
    int        BR_ROIx = 0, BR_ROIy = 0;

    int        Top_0x = 0;
    int        Top_1x = 0;
    int        Top_2x = 0;
    int        Top_3x = 0;

    int        Left_0y = 0;
    int        Left_1y = 0;
    int        Left_2y = 0;
    int        Left_3y = 0;

    int        fly01x = 0, fly01y = 0;
    int        fly02x = 0, fly02y = 0;
    int        fly03x = 0, fly03y = 0;
    int        fly04x = 0, fly04y = 0;
    int        fly05x = 0, fly05y = 0;
    int        fly06x = 0, fly06y = 0;
    int        fly07x = 0, fly07y = 0;
    int        fly08x = 0, fly08y = 0;
    int        fly09x = 0, fly09y = 0;

    int        counter = 0;

    if (argc ==2 && (capture = cvCreateFileCapture(argv[1])) != 0) {

        cout << "Analysis starting at frame (i.e. 1): ";
        cin >> start_frame;

        cout << "Total frames for analysis (i.e. 9000): ";
        cin >> total_frames;

        cout << "Analysis Step (1-15): ";
        cin >> analysis_step;

        cout << "Tracking Tail Length (1-10): ";
        cin >> tail_length;

        cout << "Enter ROI Top Left x Axis Value (in pixel): ";
        cin >> TL_ROIx;
```

```
cout << "Enter ROI Top Left y Axis Value (in pixel): ";
cin >> TL_ROIy;

cout << "Enter ROI Bottom Right x Axis Value (in pixel): ";
cin >> BR_ROIx;

cout << "Enter ROI Bottom Right y Axis Value (in pixel): ";
cin >> BR_ROIy;

Top_0x = 10;
Left_0y = 10;
Top_3x = abs(BR_ROIx - TL_ROIx) - Top_0x;
Left_3y = abs(BR_ROIy - TL_ROIy) - Left_0y;

Top_1x = (int)(Top_0x + (Top_3x - Top_0x)/3);
Top_2x = (int)(Top_0x + 2*(Top_3x - Top_0x)/3);

Left_1y = (int)(Left_0y + (Left_3y - Left_0y)/3);
Left_2y = (int)(Left_0y + 2*(Left_3y - Left_0y)/3);

background = cvLoadImage("Background.jpg", 0);
cvSetImageROI(background, cvRect(TL_ROIx, TL_ROIy, abs(BR_ROIx - TL_ROIx), abs(BR_ROIy - TL_ROIy)));

flytrack = cvCloneImage(background);
frame = cvCloneImage(background);
cvZero(flytrack);
cvZero(frame);

cvNamedWindow("FlyTrack",1);

CvMemStorage* cstorage = NULL;
CvSeq*        contours = NULL;

CvMemStorage* pstorage = NULL;
pstorage = cvCreateMemStorage(0);

CvSeqWriter   writer;
CvSeq*        psequence = NULL;

cvStartWriteSeq(CV_32SC2, sizeof(CvSeq), sizeof(CvPoint), pstorage, &writer);
psequence = cvEndWriteSeq(&writer);

cvStartAppendToSeq(psequence, &writer);

CvMoments moments;
double M00, M01, M10;
CvPoint centers;
CvMat mat;

CvFont font;
cvInitFont(&font, CV_FONT_HERSHEY_PLAIN, 1, 2, 0, 1, 8);

ofstream myfile;
myfile.open("Results.txt");

for (int i = 0; i < start_frame; i++)
{
    pframe = cvQueryFrame(capture);
}

for( int i = start_frame; i <= start_frame + total_frames; i++) {

    pframe = cvQueryFrame(capture);
    counter ++;

    if (counter == analysis_step) {
```

```
cvSetImageROI(pframe, cvRect(TL_ROIx, TL_ROIy, abs(BR_ROIx - TL_ROIx), abs(BR_ROIy - TL_ROIy)));

cvCvtColor(pframe, frame, CV_BGR2GRAY);

cvAbsDiff(frame, background, flytrack);
cvThreshold(flytrack, flytrack, 60, 255, CV_THRESH_BINARY);

cvMorphologyEx(flytrack, flytrack, 0, 0, CV_MOP_OPEN, 1);
cvMorphologyEx(flytrack, flytrack, 0, 0, CV_MOP_CLOSE, 1);

cvErode(flytrack, flytrack, 0, 1);
cvDilate(flytrack, flytrack, 0, 3);

cvSmooth(flytrack, flytrack, CV_MEDIAN, 3, 3);

cvRectangle(pframe, cvPoint(Top_0x, Left_0y), cvPoint(Top_3x,Left_3y), cvScalar(0, 255, 0), 2);

cvLine(pframe, cvPoint(Top_0x,Left_1y), cvPoint(Top_3x,Left_1y), cvScalar(0, 255, 0), 2);
cvLine(pframe, cvPoint(Top_0x,Left_2y), cvPoint(Top_3x,Left_2y), cvScalar(0, 255, 0), 2);

cvLine(pframe, cvPoint(Top_1x,Left_0y), cvPoint(Top_1x,Left_3y), cvScalar(0, 255, 0), 2);
cvLine(pframe, cvPoint(Top_2x,Left_0y), cvPoint(Top_2x,Left_3y), cvScalar(0, 255, 0), 2);

if (cstorage == NULL) {
    cstorage = cvCreateMemStorage(0);
}
else {
    cvClearMemStorage(cstorage);
}

int Nc = cvFindContours(
    flytrack,
    cstorage,
    &contours,
    sizeof(CvContour),
    CV_RETR_EXTERNAL
    );

for (; contours != 0; contours = contours->h_next) {

    CvRect r = ((CvContour*)contours)->rect;
    cvMoments(cvGetSubRect(flytrack, &mat, r), &moments, 1);
    M00 = cvGetSpatialMoment(&moments, 0, 0);
    M10 = cvGetSpatialMoment(&moments, 1, 0);
    M01 = cvGetSpatialMoment(&moments, 0, 1);
    centers.x = (int) M10/M00 + r.x;
    centers.y = (int) M01/M00 + r.y;

    cvCircle(pframe, centers, 2, cvScalarAll(255), 1, 8, 0);

    if (centers.x > Top_0x && centers.x < Top_1x && centers.y > Left_0y && centers.y < Left_1y)
    {   cvPutText(pframe, "1", centers, &font, cvScalar(255, 0, 0));
        fly01x = centers.x; fly01y = centers.y;
        CV_WRITE_SEQ_ELEM(centers, writer);
    }
    if (centers.x > Top_0x && centers.x < Top_1x && centers.y > Left_1y && centers.y < Left_2y)
    {   cvPutText(pframe, "2", centers, &font, cvScalar(255, 0, 0));
        fly02x = centers.x; fly02y = centers.y;
        CV_WRITE_SEQ_ELEM(centers, writer);
    }
    if (centers.x > Top_0x && centers.x < Top_1x && centers.y > Left_2y && centers.y < Left_3y)
    {   cvPutText(pframe, "3", centers, &font, cvScalar(255, 0, 0));
        fly03x = centers.x; fly03y = centers.y;
        CV_WRITE_SEQ_ELEM(centers, writer);
    }


    if (centers.x > Top_1x && centers.x < Top_2x && centers.y > Left_0y && centers.y < Left_1y)
```

```
    {   cvPutText(pframe, "4", centers, &font, cvScalar(0, 255, 0));
        fly04x = centers.x; fly04y = centers.y;
        CV_WRITE_SEQ_ELEM(centers, writer);
    }
    if (centers.x > Top_1x && centers.x < Top_2x && centers.y > Left_1y && centers.y < Left_2y)
    {   cvPutText(pframe, "5", centers, &font, cvScalar(0, 255, 0));
        fly05x = centers.x; fly05y = centers.y;
        CV_WRITE_SEQ_ELEM(centers, writer);
    }
    if (centers.x > Top_1x && centers.x < Top_2x && centers.y > Left_2y && centers.y < Left_3y)
    {   cvPutText(pframe, "6", centers, &font, cvScalar(0, 255, 0));
        fly06x = centers.x; fly06y = centers.y;
        CV_WRITE_SEQ_ELEM(centers, writer);
    }


    if (centers.x > Top_2x && centers.x < Top_3x && centers.y > Left_0y && centers.y < Left_1y)
    {   cvPutText(pframe, "7", centers, &font, cvScalar(0, 0, 255));
        fly07x = centers.x; fly07y = centers.y;
        CV_WRITE_SEQ_ELEM(centers, writer);
    }
    if (centers.x > Top_2x && centers.x < Top_3x && centers.y > Left_1y && centers.y < Left_2y)
    {   cvPutText(pframe, "8", centers, &font, cvScalar(0, 0, 255));
        fly08x = centers.x; fly08y = centers.y;
        CV_WRITE_SEQ_ELEM(centers, writer);
    }
    if (centers.x > Top_2x && centers.x < Top_3x && centers.y > Left_2y && centers.y < Left_3y)
    {   cvPutText(pframe, "9", centers, &font, cvScalar(0, 0, 255));
        fly09x = centers.x; fly09y = centers.y;
        CV_WRITE_SEQ_ELEM(centers, writer);
    }

}
cvFlushSeqWriter(&writer);

if (psequence->total > Nc * tail_length)
{
    for (int j = psequence->total - Nc * tail_length - 1; j >= 0; --j)
    {
        cvSeqRemove(psequence, j);
    }
}

for (int k = 0; k < psequence->total; ++k)
{
    CvPoint* p = (CvPoint*)cvGetSeqElem(psequence, k);
    cvCircle(pframe, cvPoint(p->x, p->y), 2, cvScalar(255, 0, 255), 1, 8, 0);

}

myfile << "Frame" << i << "\t";
myfile << "1" << "\t" << fly01x << "\t" << fly01y << "\t";
myfile << "2" << "\t" << fly02x << "\t" << fly02y << "\t";
myfile << "3" << "\t" << fly03x << "\t" << fly03y << "\t";
myfile << "4" << "\t" << fly04x << "\t" << fly04y << "\t";
myfile << "5" << "\t" << fly05x << "\t" << fly05y << "\t";
myfile << "6" << "\t" << fly06x << "\t" << fly06y << "\t";
myfile << "7" << "\t" << fly07x << "\t" << fly07y << "\t";
myfile << "8" << "\t" << fly08x << "\t" << fly08y << "\t";
myfile << "9" << "\t" << fly09x << "\t" << fly09y;
myfile << endl;

cvShowImage("FlyTrack",pframe);

cout << "Frame #"<< i << " has been analyzed." << endl;

counter = 0;
```

```
                cvWaitKey(30);
            }

        }

        cvEndWriteSeq(&writer);

        cvWaitKey(0);

        myfile.close();
        cvReleaseMemStorage(&cstorage);
        cvReleaseMemStorage(&pstorage);
        cvReleaseImage(&background);
        cvReleaseImage(&flytrack);
        cvReleaseImage(&frame);

        cvDestroyWindow("FlyTrack");
    }

    cvReleaseCapture(&capture);

    return 0;

}
```
