# Supplementary material for: Locomotion Induced by Spatial Restriction in Adult Drosophila
Source: PLoS One. 2015 Sep 9;10(9):e0135825. doi: 10.1371/journal.pone.0135825 (PMC4564261; doi:10.1371/journal.pone.0135825)
Supplement: S1 Appendix — (ZIP) [file pone.0135825.s002.zip › BgLearning.pdf]

```cpp
/*********************************************************
BgLearning.cpp
*********************************************************/

#include <iostream>        // for standard I/O
#include <sstream>         // string to number conversion
#include <string>          // for string
#include <cv.h>
#include <highgui.h>       // OpenCV Window I/O
#include <cxcore.h>        // Basic OpenCV structures (cv::Mat, Scalar)
using namespace std;

int main(int argc, char** argv)
{
    IplImage *pframe = NULL;
    IplImage *background = NULL;

    CvCapture *capture = NULL;

    int bg_learn_step = 0;
    int frames_for_learning;
    int counter = 0;

    if (argc ==2 && (capture = cvCreateFileCapture(argv[1])) != 0) {

        cout << "Input Background Learning Step (e.g. 10-100): ";
        cin >> bg_learn_step;

        cout << "Frame Numbers for Learning (e.g. 100-9000): ";
        cin >> frames_for_learning;

        pframe = cvQueryFrame(capture);
        background = cvCloneImage(pframe);

        cvNamedWindow("Background Learning",1);

        for(int i=1; i <= frames_for_learning; i++) {

            pframe = cvQueryFrame(capture);
            counter ++;

            if (counter == bg_learn_step) {

                cvMax(pframe, background, background);
                cvShowImage("Background Learning",background);
                cout << "Frame #"<< i << " has been learned." << endl;

                counter = 0;

                cvWaitKey(30);
            }

        }

    }

    cvWaitKey(0);

    string MyStr = string(argv[1]);
    string pfilename = MyStr.substr(0, MyStr.length() - 4) + "_Background.jpg";
    const char* filename = pfilename.c_str();

    cvSaveImage(filename,background,0);

    cvReleaseCapture(&capture);
    cvReleaseImage(&background);
    cvDestroyWindow("Background Learning");
```

```
    return 0;
}
```
